# A Genetically Encoded Isonitrile Lysine for Orthogonal Bioorthogonal Labeling Schemes

**DOI:** 10.3390/molecules26164988

**Published:** 2021-08-18

**Authors:** Ágnes Szatmári, Gergely B. Cserép, Tibor Á. Molnár, Bianka Söveges, Adrienn Biró, György Várady, Edit Szabó, Krisztina Németh, Péter Kele

**Affiliations:** 1 Chemical Biology Research Group, Institute of Organic Chemistry, ELKH Research Centre for Natural Sciences, Magyar Tudósok Krt 2, H-1117 Budapest, Hungary; cserep.balazs.gergely@ttk.hu (G.B.C.); molnartibor06@gmail.com (T.Á.M.); soveges.bianka@ttk.hu (B.S.); biro.adrienn98@gmail.com (A.B.); 2 Molecular Cell Biology Research Group, Institute of Enzymology, ELKH Research Centre for Natural Sciences, Magyar Tudósok Krt 2, H-1117 Budapest, Hungary; varady.gyorgy@ttk.hu (G.V.); szabo.edit@ttk.hu (E.S.)

**Keywords:** non-canonical amino acid (ncAA), genetic code expansion (GCE), orthogonal-bioorthogonal, dual colour labeling, self-labeling peptide tag (SLPT), HaloTag

## Abstract

Bioorthogonal click-reactions represent ideal means for labeling biomolecules selectively and specifically with suitable small synthetic dyes. Genetic code expansion (GCE) technology enables efficient site-selective installation of bioorthogonal handles onto proteins of interest (POIs). Incorporation of bioorthogonalized non-canonical amino acids is a minimally perturbing means of enabling the study of proteins in their native environment. The growing demand for the multiple modification of POIs has triggered the quest for developing orthogonal bioorthogonal reactions that allow simultaneous modification of biomolecules. The recently reported bioorthogonal [4 + 1] cycloaddition reaction of bulky tetrazines and sterically demanding isonitriles has prompted us to develop a non-canonical amino acid (ncAA) bearing a suitable isonitrile function. Herein we disclose the synthesis and genetic incorporation of this ncAA together with studies aiming at assessing the mutual orthogonality between its reaction with bulky tetrazines and the inverse electron demand Diels–Alder (IEDDA) reaction of bicyclononyne (**BCN**) and tetrazine. Results showed that the new ncAA, bulky-isonitrile-carbamate-lysine (**BICK**) is efficiently and specifically incorporated into proteins by genetic code expansion, and despite the slow [4 + 1] cycloaddition, enables the labeling of outer membrane receptors such as insulin receptor (IR) with a membrane-impermeable dye. Furthermore, double labeling of protein structures in live and fixed mammalian cells was achieved using the mutually orthogonal bioorthogonal IEDDA and [4 + 1] cycloaddition reaction pair, by introducing **BICK** through GCE and **BCN** through a HaloTag technique.

## 1. Introduction

An ever-growing demand of life science research is to be able to study multiple subcellular structures and events in their natural ambience, *in cellulo*. In an era of previously unseen resolution limits offered by a continuously expanding palette of super-resolution microscopy techniques, it is chemical biology that provides biologists with the suitable biocompatible chemistry and chemicals that enable site-specific manipulation of the biomolecules of interest, e.g., with small-synthetic fluorescent probes.

Amongst biomolecular manipulation strategies, fusion of engineered fluorescent proteins (e.g., GFP, mCherry) or tags (e.g., Halo, CLIP, SNAP) to the protein of interest represent routinely accomplished labeling methods. While both of these techniques offer highly selective means of probe installation, the size of these fusion tags often perturbs certain characteristics (mobility, function etc.) of the protein of interest (POI). Furthermore, precise localization of the POI by fusion tags is also impaired due to linkage error. Point mutations with non-canonical amino acids (ncAAs) carrying, e.g., a bioorthogonal function, are efficiently carried out using genetic code expansion techniques. Proteins carrying such a bioorthogonalized amino acid are efficiently and selectively modified with fluorescent or fluorogenic probes bearing the complementary bioorthogonal function. Such small alterations to these point mutations mean they less likely hamper the function and characteristics of the POI. The most established means for the extension of the genetic code to site-specifically incorporate ncAAs is effected by the suppression of the Amber STOP codon (UAG), but the use of other STOP codons in mammalian cells [1], or via quadruplet codons [2,3], has been described as well.

The quest for bioorthogonal chemistries that enable multi-colour bioorthogonal labeling schemes of distinct structures includes the search for mutually orthogonal-bioorthogonal reactions that can be run parallel to each other without any interfering reactions within living cells. Simultaneous incorporation of mutually orthogonal bioorthogonal moieties into distinct biomolecules (e.g., proteins) poses further challenges.

There is an emerging array of bioorthogonal click-reactions; however, only a handful of these have been shown to be mutually orthogonal, as reviewed by Smeenk et al., recently [4]. In most cases, differences in rates of otherwise similar reactions are the source of mutual orthogonality, which hampers concurrent administration of reagents. Indeed, these mutual orthogonal reactions require sequentially driven modification schemes. For example, in our pioneering work, a strain-promoted azide-alkyne cycloaddition, followed by its copper catalyzed version, effected dual labeling of alkyne and cyclooctyne modified peptides and proteins [5,6]. Nikić et. al. reported on a pulse-chase experiment where a *trans*-cyclooctene (TCO) and a cyclooctyne-modified ncAA was implemented to insulin receptors (IR) using a promiscuous tRNA/RS system accepting both amino acids, resulting in differently functionalized IR populations in the plasma membrane. Such distinctly functionalized receptors were then modified with a less reactive tetrazine to consume all the TCO. Subsequent addition of a more reactive tetrazine then led to selective modification of the cyclooctynylated insulin receptors [7]. Prescher and co-workers used sterically fine-tuned cyclopropenes, which allowed mutually orthogonal Diels–Alder and dipolar cycloadditions in combination with tetrazines and nitrile imines. Very recently, Tu et al. [8] disclosed a study on sterically demanding isonitriles, which had preferential reactivity towards sterically demanding tetrazines.

We wished to expand the toolbox of genetically encodable, bioorthogonalized amino acids enabling mutually orthogonal bioorthogonal labeling schemes. The [4 + 1] cycloaddition of sterically hindered isonitriles with sterically demanding tetrazines seemed a favorable choice for our purposes. We assumed that the isonitrile group would make an excellent functionality to add to an ncAA, because it consists of only two atoms. Thus, such an ncAA is likely to be incorporated by one of the previously reported, more promiscuous tRNA/tRNA synthetase (RS) pairs like NESPylRS^AF^/tRNA^Pyl^_CUA_ [9]. The isonitrile function is stable under physiological conditions and is non-toxic for mammals [10]. Moreover, it is not present in higher eukaryotes, and only occurs in a few natural products (e.g., in marine sponges). During the course of our work, Chen et al. [11] reported site-specific incorporation of primary and tertiary isocyanide-containing unnatural amino acids into proteins by genetic code expansion. They achieved efficient fluorogenic labeling by BODIPY-tetrazine derivatives and demonstrated a protein decaging experiment by the use of the [4 + 1] cycloaddition.

Prompted by these considerations, we designed an isonitrile-appending lysine building block, where the isonitrile moiety is linked to the *ε*-amino function of Lys through a carbamate bond. Former reports suggest that the carbamate linkage is preferred by the tRNA synthetases leading to increased incorporation efficiency. Upon successful ncAA incorporation, we wished to elaborate mutual orthogonality of the isonitrile-ncAA with cyclooctynylated proteins in dual colour-labeling schemes. To evaluate orthogonality we proposed to use a hybrid approach to achieve double bioorthogonal labeling of two distinct subcellular structures. We sought to genetically incorporate the novel isonitrile-bearing ncAA by genetic code expansion technology. To incorporate a second bioorthogonal handle into a different POI, we chose to take advantage of a fusion tag (i.e., HaloTag) capable of specifically anchoring a BCN-functionalized HaloTag substrate.

Our aim was to demonstrate the mutually orthogonal bioorthogonal labeling potential of the [4 + 1] cycloaddition of the tertiary isonitrile group with bulky tetrazines and the inverse electron demand Diels–Alder (IEDDA) reaction between (1R,8S,9s)-bicyclo [6.1.0]non-4-yn-9-ylmethanol, **BCN** and a less hindered tetrazine in vitro and *in cellulo*.

## 2. Results

### 2.1. Synthesis of Bulky Isonitrile-Carbamate-Lysine (BICK), Probes and Selectivity Studies

**Scheme 1:**
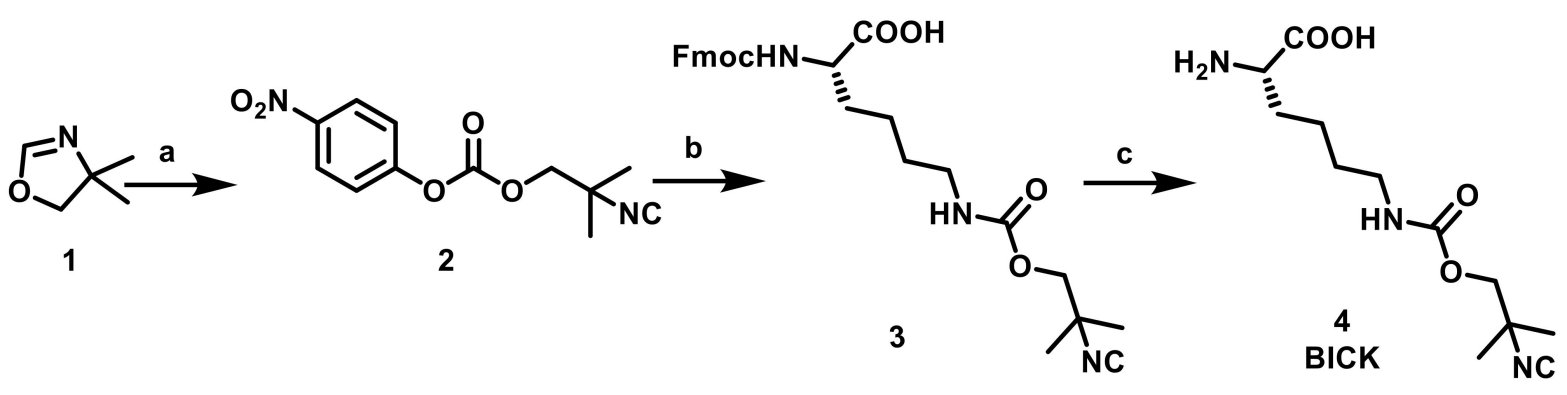
Synthesis of the bulky isonitrile-carbamate-lysine ncAA. (**a**) 4-nitrophenyl-chloroformate, *n*-BuLi, THF, −78 °C, N_2_, 1.5 h, 67%; (**b**) Fmoc-Lys-OH, DMF, DIPEA, 25 °C, 1 h, 94%; (**c**) piperidine, CH_2_Cl_2_, 25 °C, 20 min, 15%.

The target lysine derivative was prepared via a concise synthetic route outlined in Scheme 1. First, an activated isonitrile residue was synthesized in a reaction of 4,4-dimethyl-2-oxazoline (**1**) with *p*-nitrophenyl-chloroformate, using *n*-BuLi as a base. The in situ-formed isonitrile-alkoxide was instantly converted to carbonate **2** with nitrophenyl-chloroformate. Subsequently, carbonate **2** was allowed to react with Fmoc-protected lysine at its free ε-amino group to furnish protected amino acid **3**. Treatment of **3** with piperidine afforded target amino acid **4** (bulky isonitrile-carbamate lysine, **BICK**) in an acceptable yield.

**Scheme 2:**
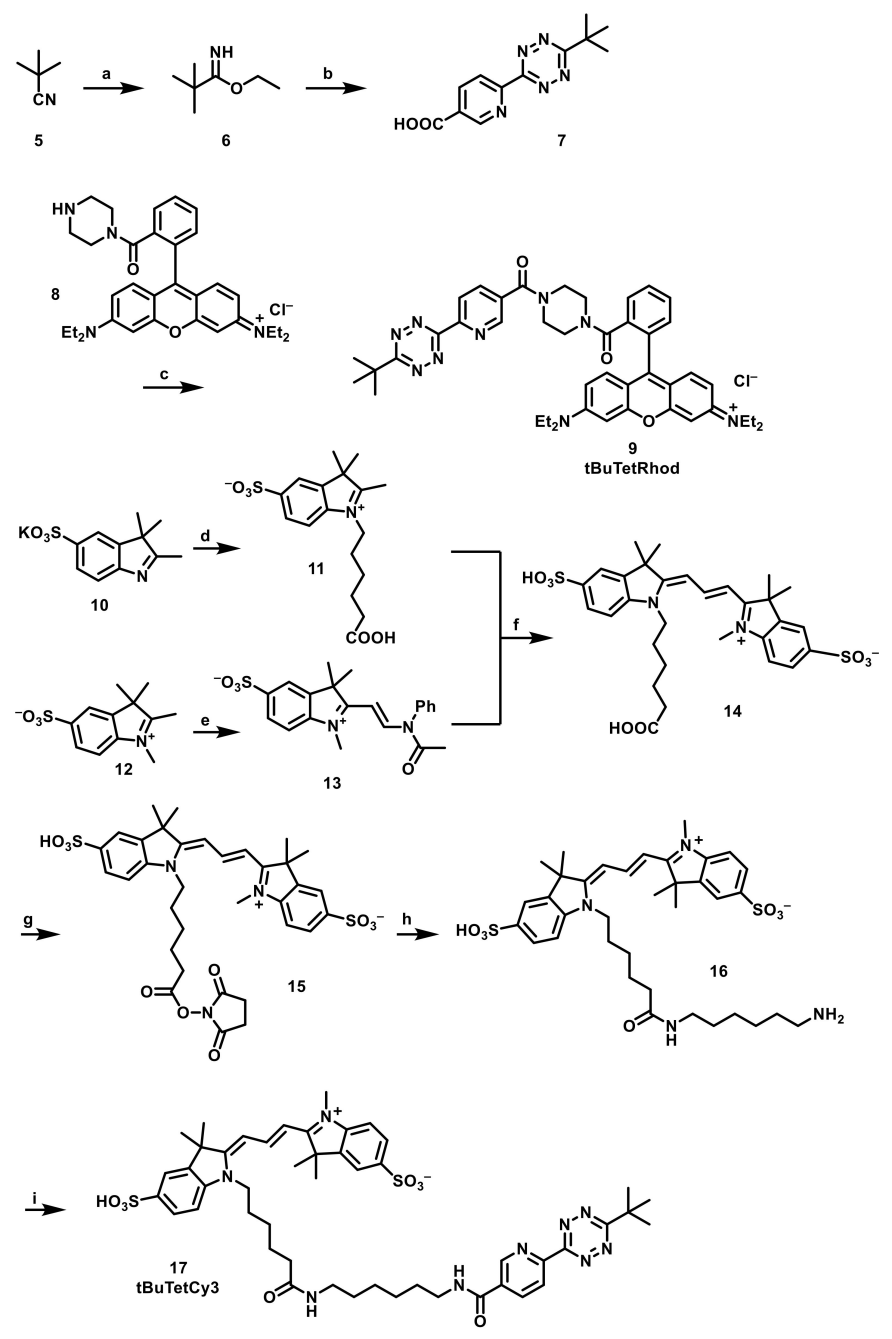
Synthesis of the sterically demanding tetrazine-rhodamine (**tBuTetRhod**) and tetrazine-sulfoCy3 (**tBuTetCy3**) probes. (**a**) EtOH, AcCl, 0 °C, 16 h, 63%; (**b**) *1.* 6-cyanonicotinic acid, N_2_H_4_ × H_2_O, S_8_, 90 °C, 1 h; *2.* (diacetoxyiodo)benzene, CH_2_Cl_2_, 25 °C, 16 h, 84%; (**c**) HBTU, HOBt × H_2_O, DIPEA, MeCN, 25 °C, 4 h, 29%; (**d**) 6-bromohexanoic acid, KI, dichlorobenzene, 150 °C, 48 h, N_2_, 40%; (**e**) *N*,*N*′-diphenylformamidine, AcOH, Ac_2_O, 120 °C, 1 h, N_2_; (**f**) pyridine, Ac_2_O, 25 °C, 16 h, 16% (2 steps); (**g**) *N*-hydroxysuccinimide, EDC × HCl, CH_2_Cl_2_, 25 °C, 2 h, (**h**) hexane-1,6-diamine, DMF, 25 °C, 16 h, 42% (2 steps); (**i**) **7**, HBTU, HOBt × H_2_O, EDIPA, MeCN, DMF, 25 °C, 1,5 h, 26%.

As of a complementary function, which is reactive towards **BICK**, we needed sterically demanding tetrazine modified probes. Thus, we needed a *t*-Bu-tetrazine that could be easily installed onto various fluorescent probes. Formerly, we have reported on nicotinic acid-derived tetrazines [12]. On the one hand, the nicotinic acid motif bears a carboxylic acid function allowing further modification. On the other hand, it efficiently activates tetrazines in IEDDA reactions due to its electron-withdrawing character. Therefore, we have designed sterically demanding nicotinic acid-derived tetrazine **7**, which was accessed via a synthetic route involving pivalonitrile (**5**) and cyanonicotinic acid (Scheme 2). This nicotinic acid-derived bulky tetrazine was coupled to rhodamine-piperazine **8** or sulfo-Cy3 **14** to furnish a membrane-permeable probe **tBuTetRhod** (**9**) and a membrane-impermeant **tBuTetCy3** (**17**), respectively (Scheme 2).

**Scheme 3:**
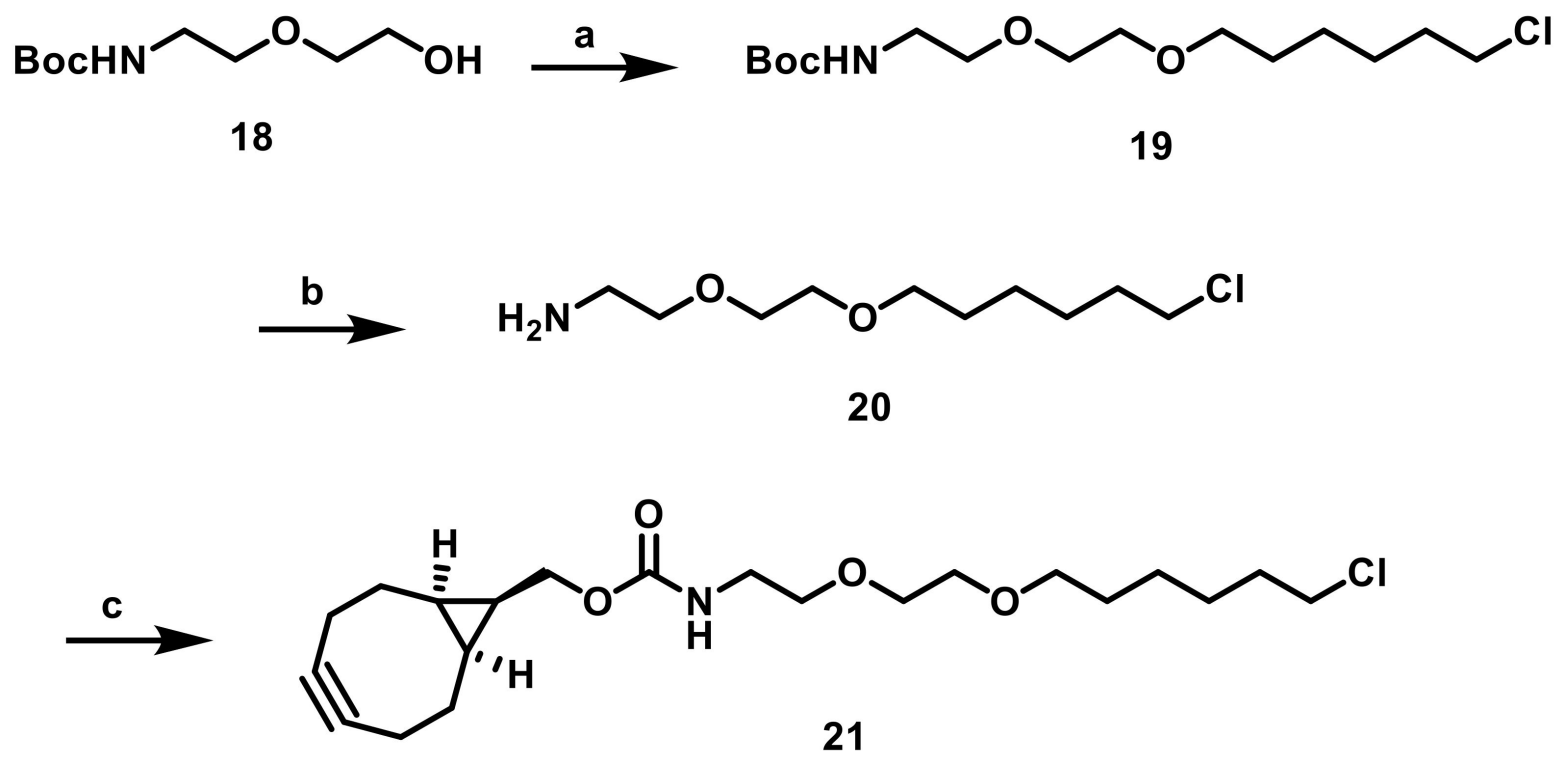
Synthesis of BCN-HaloTag substrate. (**a**) NaH, 1-chloro-6-iodohexane, THF/CH_2_Cl_2_, 0–25 °C, 16 h, 67%; (**b**) TFA/CH_2_Cl_2_ (H_2_O), 0–25 °C, 2 h, 90%; (**c**) BCN-NHS, DIPEA, MeCN, 25 °C, 16 h, 92%.

For the preparation of the **BCN-HaloTag** substrate (**21**), *N*-Boc-2-(2-hydroxyethoxy)-ethylamine (**18**) was alkylated with 1-chloro-6-iodohexane, in the presence of NaH to give **19**. Removal of the Boc-protecting group was effected by TFA to yield the free amine, **20**. Finally, compound **20** was reacted with BCN-NHS (see Supplementary Materials) using DIPEA as a base to get BCN-HaloTag substrate, **21** (Scheme 3).

**Scheme 4:**
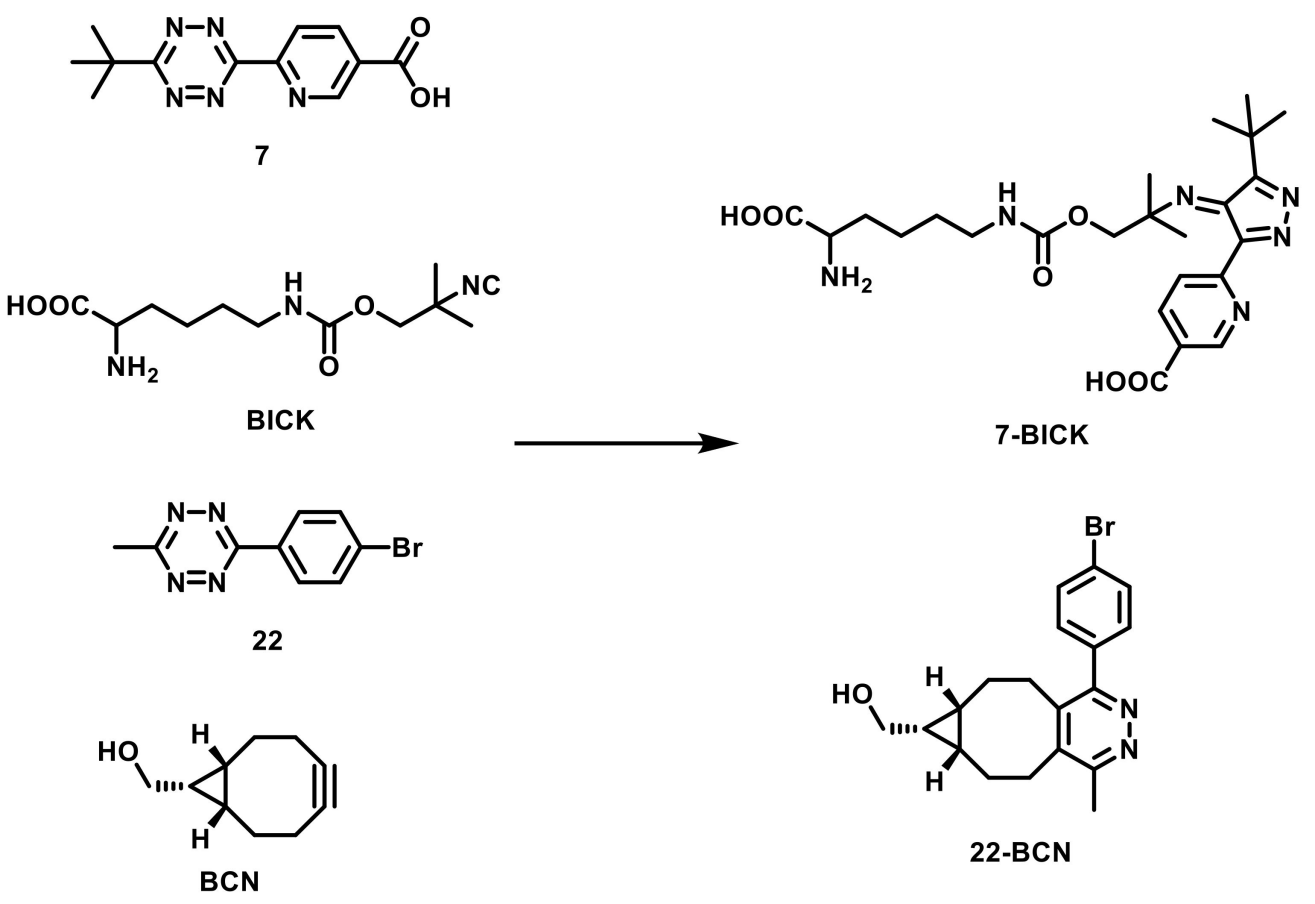
Reaction products of sterically different tetrazines with bulky isonitrile and cyclooctyne reaction partners.

In a preliminary experiment, we wished to see whether tetrazines with different steric demand react selectively with a bulky isonitrile and a cyclooctyne. To this end, we have combined tetrazines **7** and **22**, with our bulky isonitrile-carbamate lysine (**BICK**) and **BCN** (Scheme 4). Gratifyingly, LC-MS analysis of the product mixture indicated that only two products corresponding to the adducts of **7** + **BICK** and **22** + **BCN** were formed (Supplementary Materials Figure S1), suggesting mutual orthogonality of the concurrent reactions.

### 2.2. Specificity of Protein Labeling In Vitro

To test mutual orthogonality of the cycloaddition of the bulky isonitrile group and the sterically demanding *tert*-butyl-tetrazine versus the IEDDA reaction to label proteins, we selected Transferrin (TF, 76 kDa) and Bovine Serum Albumin (BSA, (66 kDa) because they are comparable in size, but still can be fully resolved on SDS-PAGE gels. We conjugated a 70 μM solution of each protein with a 5-fold excess of **NHS-NC** or **NHS-BCN**. After removing the unreacted reagents using a Sephadex G-25 spin column, we added a 500 μM solution of **Tet-SiR** and **tBuTetRhod** either separately or together to different combinations of bioorthogonalized, i.e., isonitrile- or BCN-modified, proteins. Following a 2-h incubation, the proteins were worked up using Sephadex G-25, and were immediately run on an 8% SDS-polyacrylamide gel.

**Figure 1:**
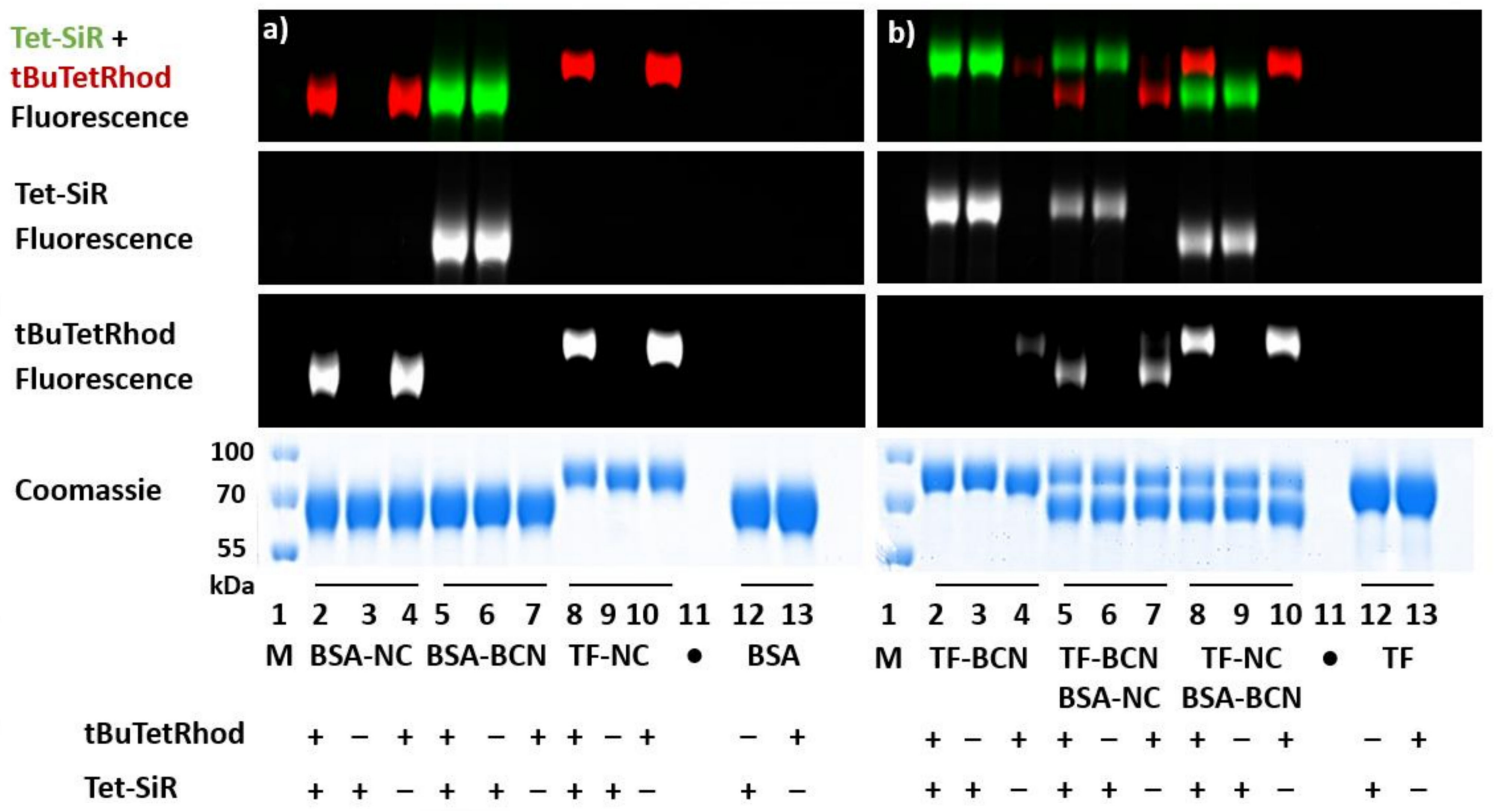
Bioorthogonal labeling of serum proteins Transferrin (TF) and Bovine Serum Albumin (BSA) to test mutual orthogonality between IEDDA and [4 + 1] cycloaddition represented by in-gel fluorescence imaging of SDS-PAGE gels. TF and BSA were modified with **NHS-NC** and **NHS-BCN**. Top images are overlays of the **Tet-SiR** and **tBuTetRhod** channels. Images in the middle two rows are the individual fluorescent channels, Coomassie staining is found in the lower row. Molecular weight corresponding to the three visible bands of the marker are indicated. Panels (**a**,**b**) represent the two gels that contain all combinations of labeling reactions. All reactions were run parallel and both of the gels in (**a**,**b**) were run together in the same tank.

The results indicate that **Tet-SiR** reacted specifically with **BCN** in either combination. Reaction of **tBuTetRhod** with isonitrile, on the other hand, was partially specific, i.e., no cross reaction was observed with **BSA-BCN**, while a faint but visible band appeared with **TF-BCN** (Figure 1b, lane 4). Cross labeling was also present when a mixture of **TF-BCN** and **BSA-NC** was treated with **tBuTetRhod** alone. However, when both bioorthogonalized proteins and both dyes were present, the two reactions were specific in each combination (Figure 1b lanes 5 and 8), indicating that cross reactivity is only present when the **BCN** moiety is not consumed in the fast IEDDA reaction. Gratifyingly, no unspecific binding of any of the dyes to any of the proteins was detected in the absence of **NC** and **BCN** (Figure 1a,b, lanes 12 and 13).

### 2.3. Genetic Incorporation of BICK into Mammalian Proteins in Cellulo

The optimized orthogonal NESPylRS^AF^/tRNA^Pyl^_CUA_ pair has been shown to efficiently translate the amber STOP codon as strained alkyne or alkene-containing ncAAs in mammalian cells with high fidelity and yield [9]. Yet, it has been shown to be flexible enough to accept further ncAAs as well [13]. Therefore, we ventured to test the ability of NESPylRS^AF^/tRNA^Pyl^_CUA_ to incorporate **BICK** into mammalian proteins.

To evaluate the incorporation efficiency and specificity of **BICK**, we transfected HEK293T cells with reporter plasmid mCherry-TAG-EGFP [14] containing an Amber STOP codon between a red (mCherry) and a green (EGFP) fluorescent reporter protein [14]. Red fluorescence was used to evaluate transfection efficiency, while green fluorescence was expected to appear selectively in cells that successfully incorporated **BICK** at the site determined by the amber STOP codon. We co-transfected this reporter construct with the NESPylRS^AF^/tRNA^Pyl^_CUA_. Following transfection, we added fresh culture medium containing 1 mM **BICK**, or, as a positive control, 0.25 mM bicyclo [6.1.0]non-4-yn-9-ylmethanol-lysine (**BCNK**), an ncAA known to be efficiently incorporated by NESPylRS^AF^ into mammalian proteins [15].

**Figure 2:**
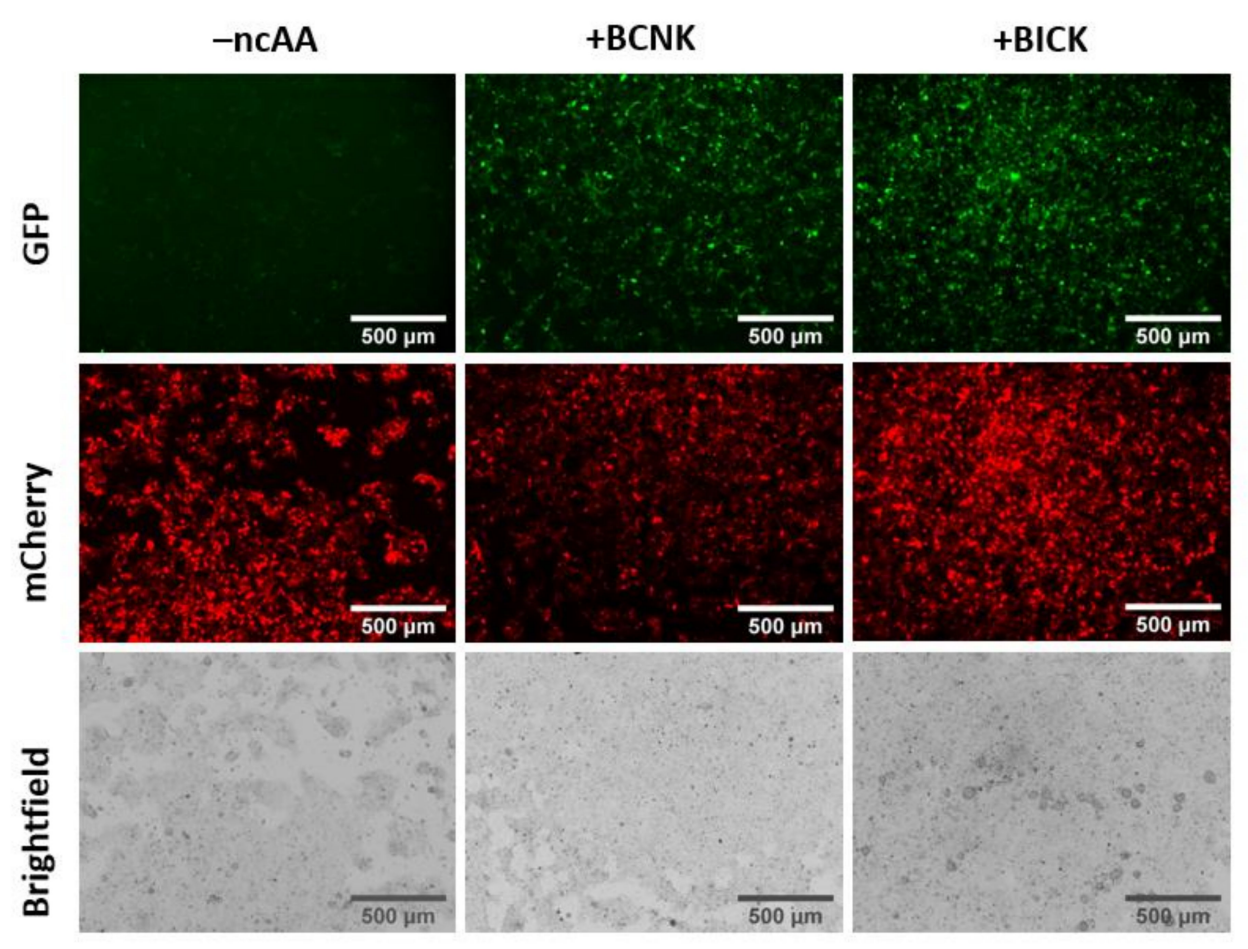
Live cell fluorescent wide field imaging of specific incorporation of 250 μM **BCNK** (**middle column**) and 1 mM **BICK** (**right column**) into pmCherry-TAG-EGFP in HEK293T cells. mCherry-positive cells were successfully transfected with the plasmid bearing the POI, while the amber STOP codon is suppressed only in GFP-positive cells by a site-specific incorporation of the added ncAAs. Scale bar = 500 μm.

**Figure 3:**
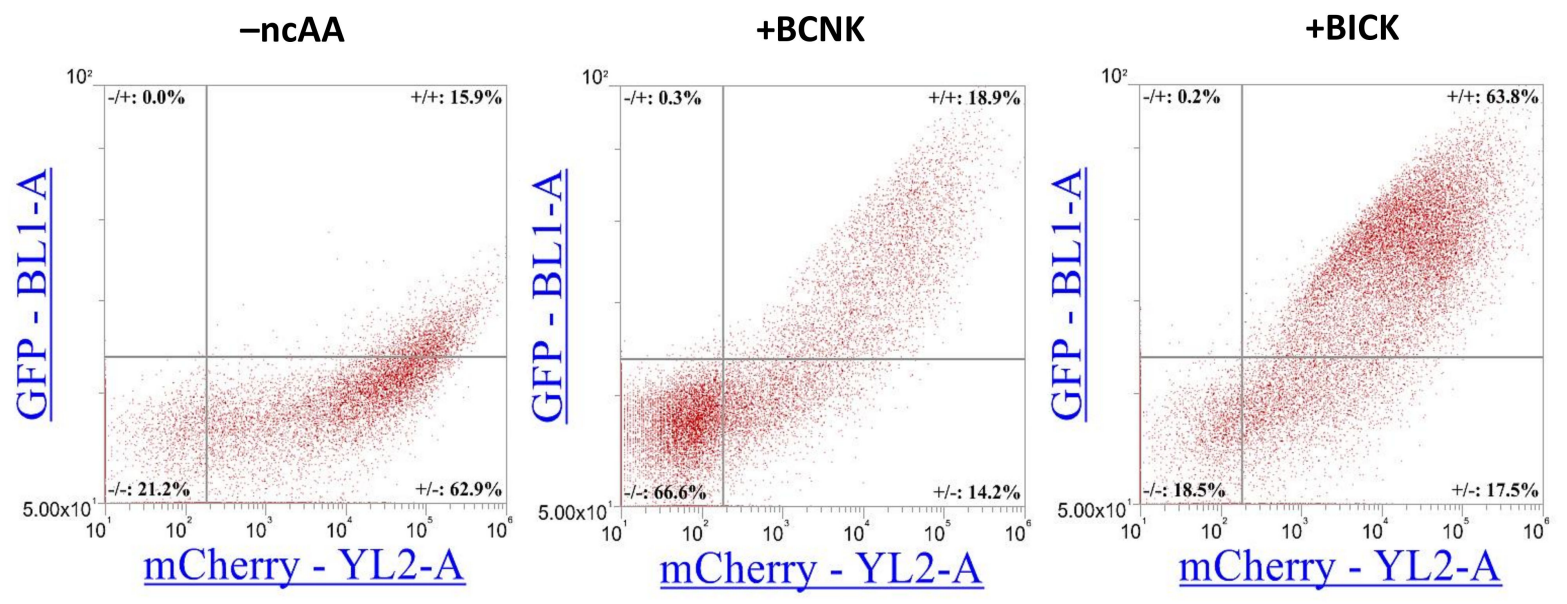
Flow cytometry analysis of the reporter pmCherry-TAG-EGFP in HEK293T cells to assess the efficiency of genetic incorporation of 1 mM **BICK** (**right**) as compared to 250 μM **BCNK** (**middle**), or no ncAA added (**left**) by NESPylRS^AF^. On the horizontal axis, fluorescence intensity corresponding to mCherry is shown, while GFP fluorescence is plotted on the vertical axis. The latter indicates successful incorporation and Amber suppression rates. Fluorescence intensity is indicated in arbitrary units.

To our delight, microscopy analysis indicated the appearance of green fluorescence in a significant number of cells alongside red fluorescence, suggesting that **BICK** is efficiently incorporated into proteins (Figure 2). Flow cytometry analysis of cells was utilized to quantify the efficiency and specificity of ncAA incorporation (Figure 3 and Supplementary Materials Figure S2). We observed that an average of 81% of transfected cells incorporated **BICK**, which is similarly efficient to the incorporation of known substrate **BCNK** (76%) and significantly higher than the negative control that contains mCherry-TAG-EGFP and NESPylRS^AF^/tRNA^Pyl^_CUA_ plasmids, but no ncAA (20%). Note that unspecific Amber suppression in the ncAA negative samples resulted in relatively low level GFP fluorescence, which is considered the result of the normal leakage of the single Amber stop codon due to non-specific incorporation of natural amino acids, causing a minor background [16]. We also showed in flow cytometry experiments that incorporation of **BICK** is concentration-dependent (Figure S2b,c). Significant incorporation of **BICK** was already detected at 0.125 mM concentration and this showed an increasing tendency towards 2 mM concentration. However, the percentage of GFP-positive (thus Amber-suppressed, with a GFP fluorescence threshold drawn at the level of the no-ncAA controls) cells did not seem to differ to a biologically significant extent above 1 mM **BICK**. We also considered the median fluorescence intensities of GFP-positive cells (Figure S2c). This was higher in the case of 2 mM **BICK**, but progression of the curve was already reaching a plateau at 2 mM. 1 mM **BICK** concentration already gives a high value, moreover, this was closest to the value measured in the case of 0.25 mM BCNK, our standard. Therefore, it seemed a reasonable concentration to apply in live cell experiments.

The MTT assay revealed that **BICK** lacked any cytotoxicity (Figure S3). The viability of HEK cells apparently remains at 100% after 16 h incubation with 1 mM **BICK**.

Stability tests (Figure S4) indicated that 1 mM **BICK** retains its full integrity for at least 48 h at 37 °C in 20 mM HEPES buffer or in HEPES completed with 2 mM GSH to model the typical cellular environment.

### 2.4. Live Cell Labeling of Insulin Receptors (IR) on the Extracellular Side of the Cell Membrane

**Figure 4:**
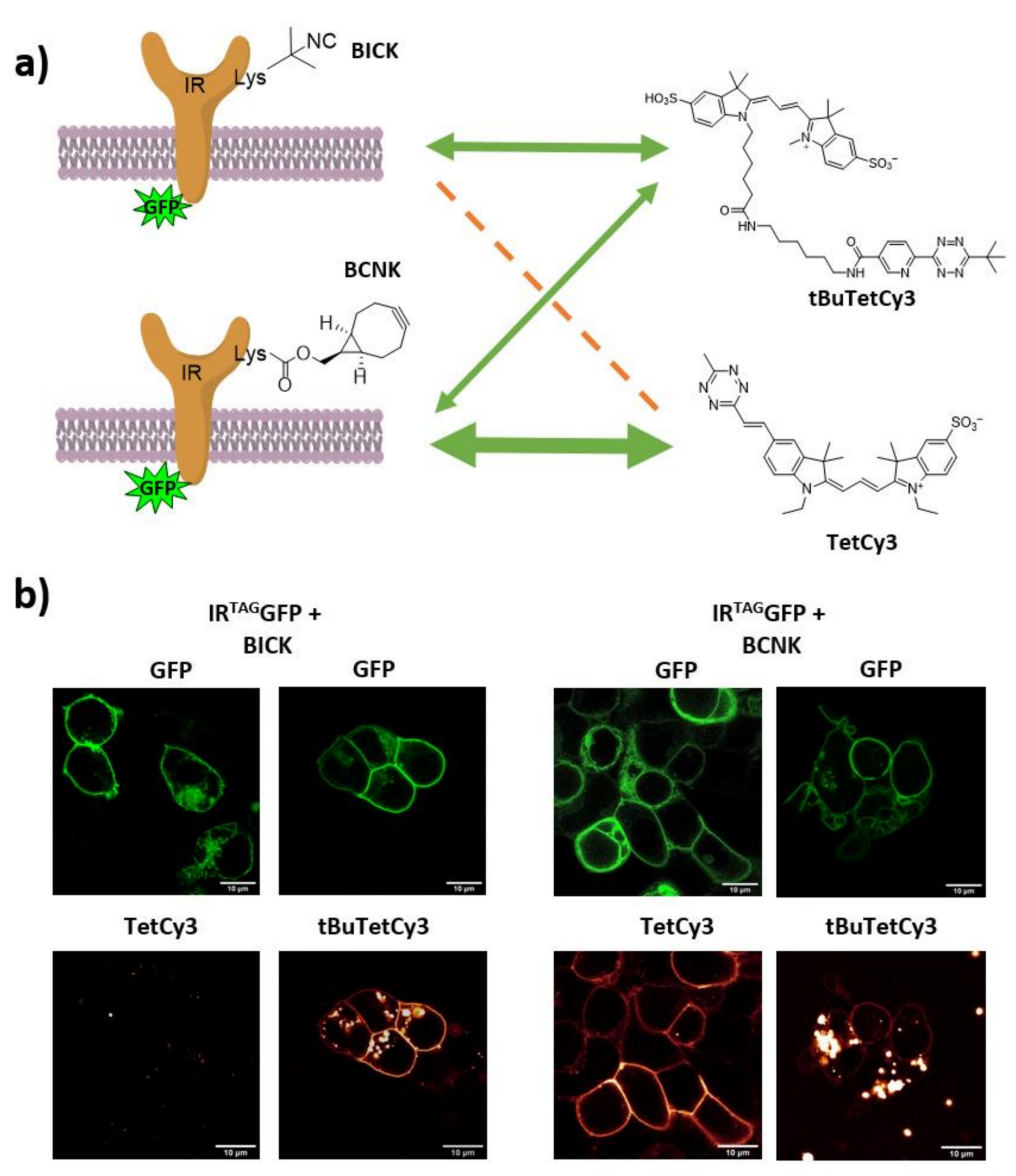
(**a**) Live cell bioorthogonal labeling scheme of overexpressed insulin receptor-GFP fusion protein (IR^TAG^GFP) in HEK293T cells with incorporation of either of the ncAAs **BICK** or **BCNK** and testing the labeling efficiency of a tertbutyl-tetrazine conjugated dye (**tBuTetCy3**) and a tetrazine conjugated dye (**TetCy3**). (**b**) Confocal images of the live cell labeling experiment outlined in the scheme in (**a**). We tested the orthogonality *in cellulo* of the bioorthogonal reactions between the ncAAs **BICK** (1 mM) or **BCNK** (250 μM) genetically incorporated into IR^TAG^GFP and labeled with **tBuTetCy3** (100 μM, overnight) or **TetCy3** (3 μM, 60 min). Detection windows: green channel (GFP) 495–550 nm; red channel (**tBuTetCy3** and **TetCy3** conjugate) 565–650 nm. False colour codes: Green: The reporter fluorophore GFP shows location of efficient IR expression. Red: Labeling of IR with **tBuTetCy3** and **TetCy3**. Scale bar = 10 μm.

Given bulky-isonitrile bearing ncAA, **BICK** was incorporated by the NESPylRS^AF^/tRNA^Pyl^_CUA_ pair site-specifically, we moved on to test whether mutual orthogonality of the cycloaddition of the bulky isonitrile group and the sterically demanding *tert*-butyl-tetrazine versus the inverse electron demand Diels–Alder reaction between **BCN** and methyl-tetrazine can be demonstrated under live-cell conditions. To this end, as indicated in Figure 4a, we incorporated either **BICK** or **BCNK** into insulin receptors (IR) bearing an Amber (TAG) mutation at an extracellular position (K676) by transfecting HEK293T cells simultaneously with plasmids coding for the IR^TAG^GFP fusion construct [7] and the NESPylRS^AF^/tRNA^Pyl^_CUA_ pair [9]. Then we carried out single colour labeling reactions on both ncAAs with *tert*-butyl-tetrazine-sulfo-Cy3 (**tBuTetCy3**, 100 μM, overnight) and Cy3-tetrazine (**TetCy3**, (**25**) described in the work of Knorr et al. [17], 3 μM, 60 min).

As expected, **TetCy3** labeling of **BCNK** was strong and specific, and reassuringly, no Cy3 signal was detected on **BICK**-bearing cells, while **tBuTetCy3** labeling was robust in this latter cell population, although some undesired intracellular particles were also detected, probably due to dye aggregation or endosomal uptake. This might have resulted from the longer labeling interval with a relatively high dye concentration needed for the efficient reaction of the bulky tetrazine under live cell conditions. This also leads to some cross labeling as a result of the reaction of **tBuTetCy3** with **BCNK** (Figure 4b). The phenomenon was not unexpected, however, as the in-gel fluorescence tests gave similar results. Based on the above findings, we reasoned that for orthogonal dual colour labeling schemes, quick saturation of the **BCN** moieties with sterically non-demanding tetrazine-dyes should be carried out first, followed by the addition of the slowly reacting **tBuTet**-dye to conjugate to **BICK** bearing proteins. Longer labeling times with **tBuTetCy3** was safe for live HEK293T cells according to the MTT test carried out under the same conditions as the live cell labeling reactions, as shown in Figure S3.

### 2.5. Dual Colour Labeling and Imaging of Subcellular Structures in Fixed Cells

**Figure 5:**
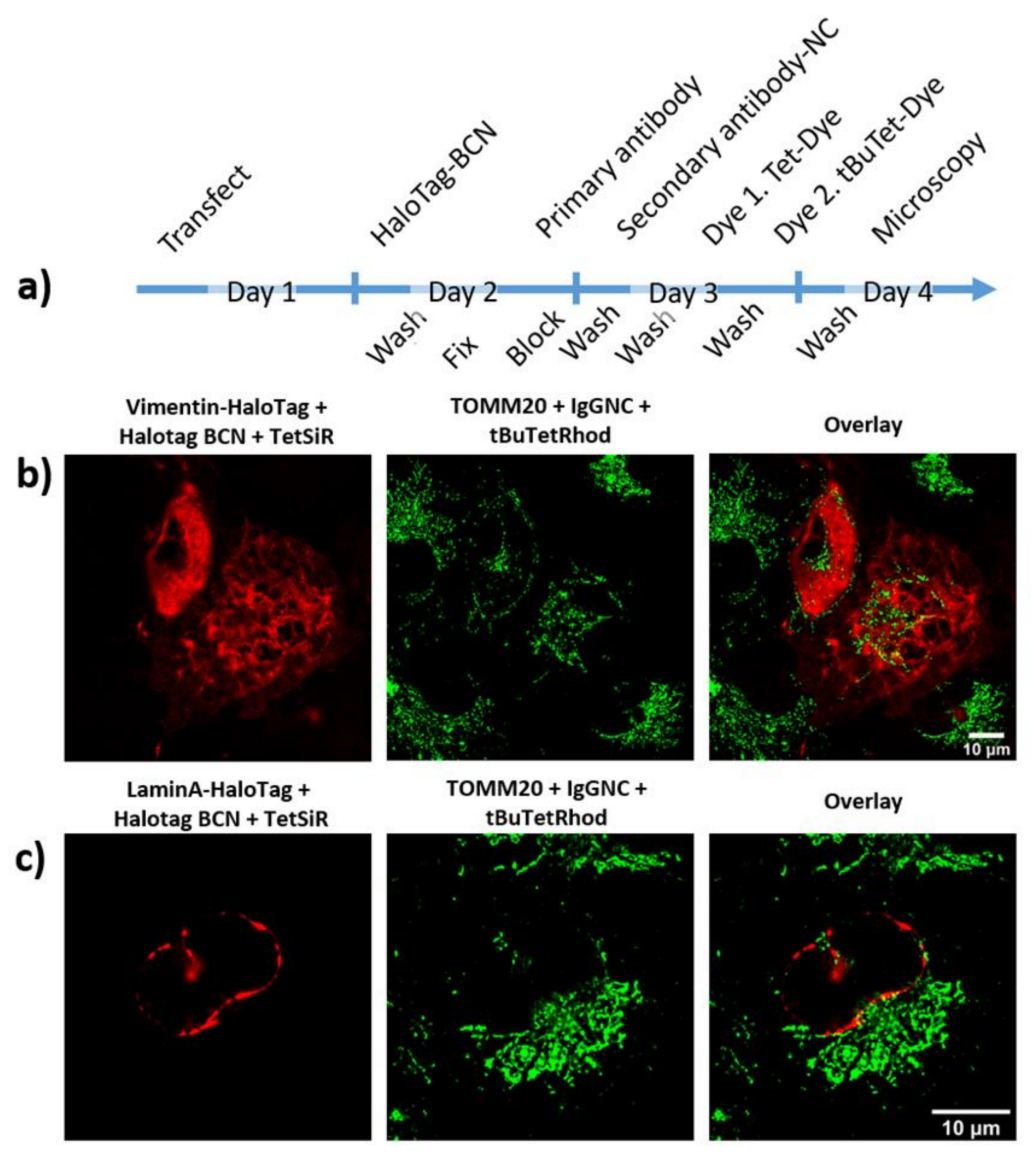
(**a**) Chart representing the workflow for fixed-cell dual-colour bioorthogonal labeling inside COS-7 cells expressing a HaloTag fused POI. Cells were fixed after the reaction with HaloTag ligand-**BCN** and labeled with **Tet-SiR** in parallel with immunofluorescent labeling of mitochondria using anti-TOMM20 primary antibody and **IgGNC** (**a**) secondary antibody activated with NHS-isonitrile (**NHS-NC**, **23**)—followed by the cycloaddition of tertbutyl-tetrazine conjugated rhodamine (**tBuTetRhod**). (**b**,**c**) Confocal images of dual colour fixed cell labeling using the strategy described in (**a**). (**b**) COS-7 cells expressing Vimentin-HaloTag construct; double labeled through the described fixed-cell labeling strategy. (**c**) COS-7 cells expressing LaminA-HaloTag; also double-labeled as described in (**a**). Detection windows: red channel (rhodamine) 565–615 nm; far red channel (**SiR**) 675–800 nm. False colours: Red: **Tet-SiR** labeling of HaloTag + HaloTag ligand-BCN tagged nuclear proteins. Green: Labeling of TOMM20 with rhodamine by cycloaddition between isonitrile/tertbutyl-tetrazine groups. Scale bar = 10 μm.

After labeling insulin receptors on the outer membrane-surface of live cells, we turned to double labeling of ultrastructures in fixed cells. We chose two intermediate filament type proteins: Vimentin (type III) and LaminA (type V)—the latter lines the inside of the nuclear membrane. The third protein, TOMM20 (Translocase Of Outer Mitochondrial Membrane 20), is a mitochondrium marker. We assumed that we would be able to label complex intracellular structures after fixation and permeabilization of cells. Since labeling of the **BCN**-reactive group is possible after fixation with formaldehyde (PFA) and Triton X-100, we could recruit the HaloTag system, i.e., an intracellular POI fused to the HaloTag self-labeling enzyme [18,19]. This allowed easy labeling of various structures such as vimentin (Figure 5b) and LaminA (Figure 5c) without the need for an antibody. The isonitrile group, on the contrary, was not preserved for labeling when administered before fixation in our hands; therefore, we used a bulky-isonitrile-modified secondary antibody (IgGNC) together with anti-TOMM20 primary antibody to label mitochondria after fixation.

Our workflow (Figure 5a) started with transfection of COS-7 cells that have more voluminous cytoplasm relative to their nucleus, when compared to HEK293T cells. We used plasmids encoding intracellular proteins (Vimentin or LaminA) fused to Halo-Tag. The **HaloTag-BCN** substrate was then added to cells one day after transfection to introduce the first bioorthogonal reaction partner. After extensive washing, cells were fixed at this point, and after further washing and blocking of unspecific antibody binding sites, TOMM20 primary antibody was allowed to bind at 4 °C overnight. Cells were washed extensively, and the isonitrile conjugated secondary antibody was added. At this point each of the two bioorthogonal reactive groups (**BCN** and isonitrile) were ready to receive their reaction partners. Knowing that the **BCN**-tetrazine reaction is fast and capable of consuming practically every **BCN** moiety, we stained the specimens with **Tet-SiR** (**24**), a methyl-tetrazine functionalized siliconrhodamine dye (described in the work of Kozma et al.) [20] first, for one hour. After extensive washing, we proceeded with staining with the sterically demanding, tertbutyl-tetrazine-rhodamine (**tBuTetRhod**) probe, which was more specific with less background on the fixed samples compared to the **tBuTetCy3** used to stain live cells. The reaction between isonitrile and tertbutyl-tetrazine groups is ca. two orders of magnitude slower than the preceding IEDDA reaction, therefore this labeling reaction was carried out overnight. After the final washes, confocal microscopy revealed successful dual-colour labeling of mitochondria and vimentin or LaminA using the orthogonal-bioorthogonal chemistry elaborated in this work.

### 2.6. Dual Colour Labeling and Imaging of Subcellular Structures in Live Cells

**Figure 6:**
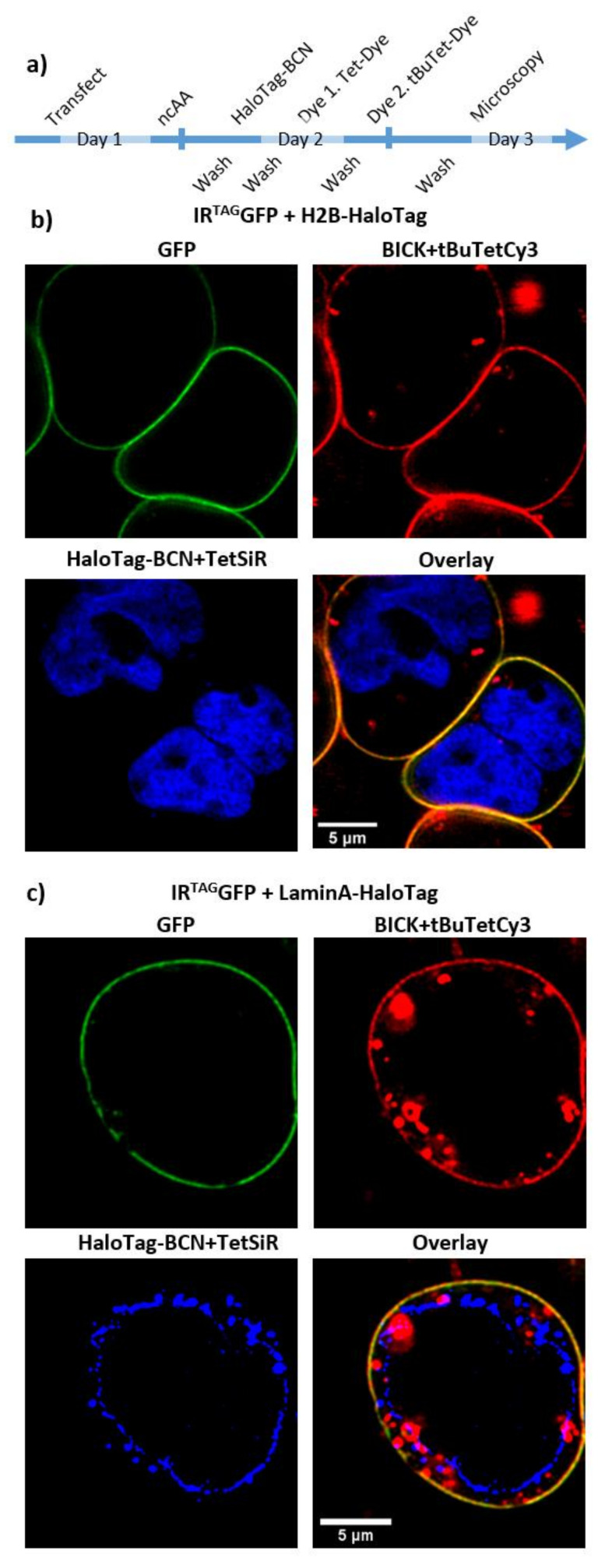
(**a**) Diagram representing the workflow for live cell dual-colour bioorthogonal labeling of overexpressed proteins in HEK293T cells combining incorporation of the ncAA **BICK** and the cycloaddition of a tertbutyl-tetrazine conjugated dye (**tBuTetCy3**) and labeling through HaloTag-BCN with **Tet-SiR**. (**b**,**c**) Confocal images of dual-colour live cell labeling with the orthogonal-bioorthogonal reactions between the ncAA **BICK** genetically incorporated into IR-^TAG^-GFP and **tBuTetCy3**, and nuclear proteins expressed in fusion with the HaloTag self-labeling enzyme labeled with **Tet-SiR** through HaloTag-BCN. (**b**) H2B-HaloTag construct; (**c**) LaminA-HaloTag construct was used. Detection windows: green channel (GFP) 495–550 nm; red channel (**tBuTetCy3** conjugate) 565–650 nm; far red channel (**SiR**) 650–800 nm. False colour codes: Green: The reporter fluorophore GFP shows location of efficient IR expression. Red: Labeling of IR with **tBuTetCy3** by cycloaddition between isonitrile/tertbutyl-tetrazine groups. Blue: Labeling of cell nucleus related proteins with **Tet-SiR** through HaloTag-BCN. Yellow in overlays: Green GFP and red **tBuTetCy3** labeling of insulin receptors result in yellow colour. Scale bar = 5 μm.

As we already incorporated **BICK** into the insulin receptor (IR) bearing an Amber STOP codon using GCE in HEK293T cells and established a live cell labeling protocol with **tBuTetCy3** (Section 2.3), we next aimed to achieve dual colour live cell labeling. To this end, we introduced the second bioorthogonal moiety (**BCN**) through a plasmid encoding an intracellular POI fused to the HaloTag self-labeling enzyme, similarly to the fixed-cell protocol. Our workflow is summarized in Figure 6a. As POIs we chose nuclear proteins, LaminA and H2B. The histone protein H2B, like LaminA, is also located in the nucleus and is involved in the structural organization of chromosomes. Following transfection of the cells with the plasmids and protein expression, **BICK** was removed and HaloTag-**BCN** substrate was added to introduce the other bioorthogonal reaction partner either to Lamin or H2B. Following removal of unreacted HaloTag-**BCN**, **Tet-SiR** was added to label the first subcellular structure. This labeling reaction is rapid, resulting in specific and background-free labeling, and consumption of the **BCN** moieties. After extensive washing of the cells, the second probe, **tBuTetCy3**, was added. The next day, after extensive washing, the medium was changed to indicator-free DMEM, and confocal microscopy imaging was carried out on the same day, without fixation. Labeling with **tBuTetCy3** was specific; however, some undesired dye accumulation in endocytotic vesicles could also be observed. Using the above-described method we could carry out efficient double bioorthogonal labeling of insulin receptors and histone H2B (Figure 6b) or LaminA (Figure 6c) in live HEK293T cells. We detected no unspecific labeling, which meant that the **BCN** moieties were fully consumed in the first step, resulting in specific labeling of H2B or LaminA at the inner lining of cell nuclei. Insulin receptors bearing an isonitrile-carbamate-lysine ncAA were also specifically labeled in the following step.

## 3. Discussion

There is an ongoing demand for dual or even multicolour labeling schemes of subcellular components in life sciences. Thus, mapping possible mutually orthogonal-bioorthogonal reactions is a key step to facilitate exploitation of the advantages of fluorescent dyes with superior photophysical properties. [21] Genetic code expansion enables site-specific incorporation of bioorthogonal handles with minimal perturbation and linkage error. Such biocompatible click reactions were successfully used to label proteins following genetic incorporation of bioorthogonalized ncAAs into POIs in *E. coli* bacteria [22] and mammalian cells, enabling protein turnover studies, using a pulse-chase strategy [7], or at different sites within a protein, enabling FRET studies, and in different cell surface proteins, allowing dual-colour labeling schemes, as well [1].

Our aim was to develop a dual colour labeling method to visualize subcellular structures using mutually orthogonal bioorthogonal reactions. To this end, we focused on the recent report of Tu and colleagues [8] reporting on the [4 + 1] cycloaddition reactions of tetrazines and isonitriles. It was reported that unlike in case of the IEDDA reactions of tetrazines and strained alkenes/alkynes, increased steric demand facilitates the [4 + 1] cycloaddition reactions of tetrazines towards sterically also demanding isonitriles, allowing selective reaction in the presence of strained alkenes (e.g., TCO or norbornene). It was also demonstrated that the tetrazine-isonitrile reaction is orthogonal to the strain-promoted reaction of azides and dibenzocyclooctyne [23]. This latter report also highlighted the importance of the size of the isonitrile function as it enabled enzymatic incorporation of a glucosamine-NC derivative into surface glycans. These findings have prompted us to investigate the orthogonality of the reaction between a bulky tetrazine and a tertiary isonitrile with a sterically less demanding tetrazine-cyclooctyne pair. We also wished to explore the possibility of incorporating a tertiary isonitrile moiety into proteins using genetic code expansion.

We hypothesized that the IEDDA reaction of the cyclooctyne **BCN** and a methyl-tetrazine might presumably be mutually orthogonal to the cycloaddition reaction of bulky isonitriles with a sterically demanding tetrazine. **BCN** has proved a useful handle to render biomolecules bioorthogonalized and allows high signal-to-noise ratios for super-resolution microscopy studies [24,25]. We have assessed the mutual orthogonality of the two reactions in solution followed by LC-MS and in vitro, using **NC** and **BCN** modified proteins in combination with tBuTetrazine and MeTetrazine modified probes. Gel electrophoretic images confirmed that the reactions are indeed mutually orthogonal, though the bulky tetrazine has some tendency to cross-react with BCN. This latter finding highlighted the limitation of the two pairs, i.e., the **BCN** moiety should be fully consumed in a reaction with MeTet-probe before adding the tBuTet probe. This suggests a sequentially executed labeling scheme.

Following this, we have synthesized a new bulky-isonitrile bearing ncAA (**BICK**) to elaborate its GCE applicability. **BICK** was found to be incorporated extremely efficiently into extra- and intracellular POIs by Amber suppression technology using the NESPylRS^AF^/tRNA^Pyl^_CUA_ pair [9].

Although examples for multiple modification of different intracellular structures via GCE exist [1,3,26], incorporation of multiple ncAAs using GCE is yet to be generalized. Therefore, we have chosen to combine GCE with self-labeling HaloTag fusion protein technique for the incorporation of **BICK** and **BCN** moiety, respectively.

The other problem that impairs ideal visualization of subcellular structures using mutually orthogonal bioorthogonal reactions is the considerable difference in the rates of the two reactions. While IEDDA is fast, the [4 + 1] cycloaddition of isonitriles and bulky tetrazines is orders of magnitude slower, being in the same range as the strain-promoted alkyne-azide cycloaddition (SPAAC) [11,27]. The slow reaction between isonitrile and bulky tetrazine in our case required elevated labeling concentrations and time, that accounted for the less efficient intracellular labeling of **BICK** tagged proteins with cell-permeant **tBuTetRhod**, due to a higher nonspecific background accumulation. Therefore, we turned towards the labeling of insulin receptors on the cell surface using a non-permeable dye, **tBuTetCy3** specifically designed for this particular task. Labeling of IR was successful in this way, although some endosomal accumulation of the dye was visible, resulting from the longer reaction interval. A task for future studies remains the design of a fluorogenic, cell-permable, easy-to-wash out tBuTet- dye to enable intracellular labeling through **BICK**.

A further adjustment of our labeling protocol was necessary due to the slight affinity of tBuTet moiety towards **BCN** as described above. The considerable difference between the reaction rates does not allow the simultaneous addition of the differently functionalized probes in live cell labeling schemes, as the much higher reagent concentration needed for the sterically demanding tetrazine-bearing fluorescent probe leads to an elevated background.

In conclusion, we presented a new, bulky isonitrile-bearing amino acid, which is found to be incorporated efficiently into proteins by the NESPylRS^AF^/tRNA^Pyl^_CUA_ pair. Results suggest that the mutually orthogonal bioorthogonal nature of isonitrile and **BCN** moieties enables double bioorthogonal labeling through [4 + 1] and IEDDA cycloaddition click reactions. To achieve mutual orthogonality in live cell conditions, a sequential protocol is required due to the limited reaction rate of the [4 + 1] cycloaddition relative to the IEDDA reaction of tetrazines and strained alkynes. This required the design of a specific, membrane-impermeable dye limiting the use of isonitrile-bulky tetrazine reactions to the extracellular labeling schemes. Bearing in mind these limitations, we have introduced a feasibly functioning mutually orthogonal bioorthogonal reaction pair, and demonstrated their use in double labeling schemes using a sequential protocol. Such mutually orthogonal chemistries could be exploited to achieve multi-colour labeling of outer membrane structures (receptors, etc.) through **BICK** incorporation via GCE, and distinct subcellular structures using, e.g., HaloTag fusion. In the future, incorporation of both members of the orthogonal pair by GCE would be a useful goal, along with improved fluorogenic tBuTet-dyes to facilitate intracellular live cell labeling through isonitrile with a low background.

## 4. Materials and Methods

All materials and methods are described in the Supplementary Materials available online at Supplementary Materials File 1.

## Data Availability

The data presented in this study are available in this article.

## Supplementary Materials

Supplementary Materials File 1, [28,29,30,31,32]. Figure S1. LC-MS chromatograms show the product peaks of the four-party reaction including tetrazines with different steric demand (**7** and **22**) with bulky isonitrile-carbamate lysine (**BICK**) and **BCN** as indicated in Scheme 4 in the main text. Figure S2. Flow cytometry analysis of the reporter pmCherry-TAG-EGFP in HEK293T cells to assess the efficiency of genetic incorporation of **BICK** as compared to **BCNK**, or no ncAA added by NESPylRS^AF^. Figure S3. MTT cell viability test of **BICK** and **tBuTetCy3** (**17**). Figure S4. Stability test of **BICK**. Text S1: Supplementary Materials and Methods.
