# Supplementary material for: A Genetically Encoded Isonitrile Lysine for Orthogonal Bioorthogonal Labeling Schemes
Source: Molecules. 2021 Aug 18;26(16):4988. doi: 10.3390/molecules26164988 (PMC8402055; doi:10.3390/molecules26164988)
Supplement: Supplementary file 1 [file molecules-26-04988-s001.zip › molecules-1259139-supplementary.pdf]

Supplementary Information

A Genetically Encoded Isonitrile Lysine for Orthogonal Bioorthogonal Labeling Schemes

Ágnes Szatmári [1,*], Gergely B. Cserép [1], Tibor Á. Molnár [1], Bianka Söveges [1], Adrienn Biró [1], György Várady [2], Edit Szabó [2], Krisztina Németh [1,*] and Péter Kele [1,*]

[1] Chemical Biology Research Group, Institute of Organic Chemistry, ELKH Research Centre for Natural Sciences, Magyar Tudósok krt 2, H-1117 Budapest, Hungary; cserep.balazs.gergely@ttk.hu (G.B.C.); molnartibor06@gmail.com (T.Á.M.); soveges.bianka@ttk.hu (B.S.); biro.adrienn98@gmail.com (A.B.)

[2] Molecular Cell Biology Research Group, Institute of Enzymology, ELKH Research Centre for Natural Sciences, Magyar Tudósok krt 2, H-1117 Budapest, Hungary; varady.gyorgy@ttk.hu (G.V.); szabo.edit@ttk.hu (E.S.)

* Correspondence: szatmari.agnes@ttk.hu (Á.S.); nemeth.krisztina@ttk.hu (K.N.); kele.peter@ttk.hu (P.K.)

1 .Supplementary Figures

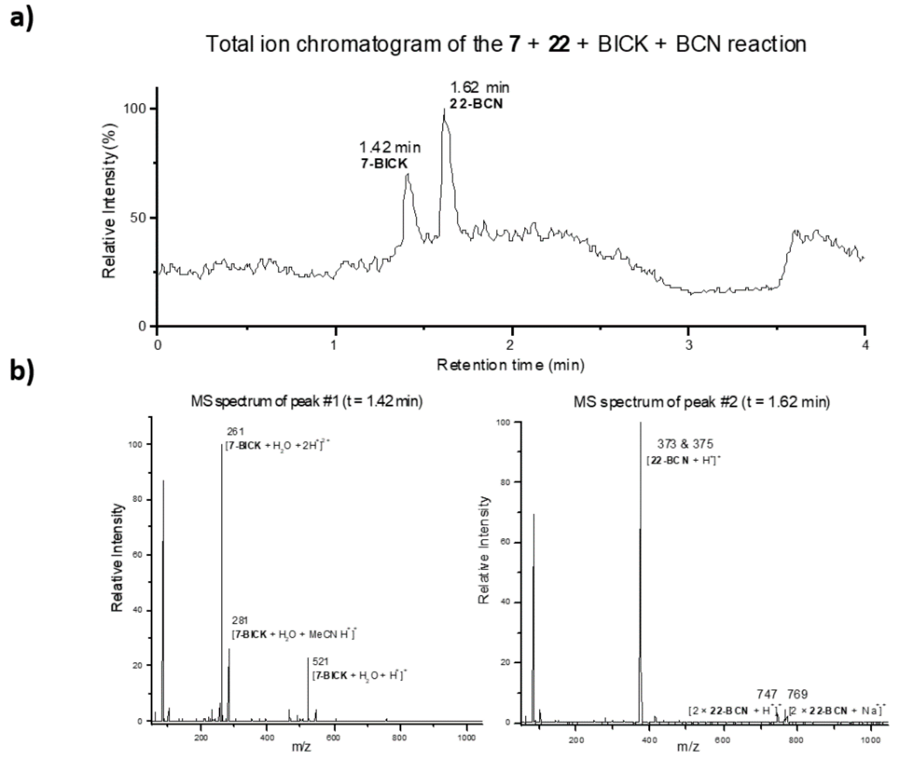

Figure S1.
LC-MS chromatograms show the product peaks of the four-party reaction including tetrazines with different steric demand (**7** and **22**) with bulky isonitrile-carbamate lysine (**BICK**) and **BCN** as indicated in Scheme 4 in the main text. The four compounds were combined in equimolar ratios, 2.5 mM each in acetonitrile:water (1:1). The reaction was run at 25 °C for 2 hours, the products were then analyzed by LC-MS. The resulting product mixture contained the two expected products exclusively suggesting mutual orthogonality of the concurrent reactions. **(a)** Total ion chromatogram of the product mixture, with the sole two products corresponding to the adducts of **7** + **BICK** and **22** + **BCN**. **(b)** MS spectra of the two peaks, supporting correct identification of the corresponding compounds as **7-BICK** and **22-BCN**.

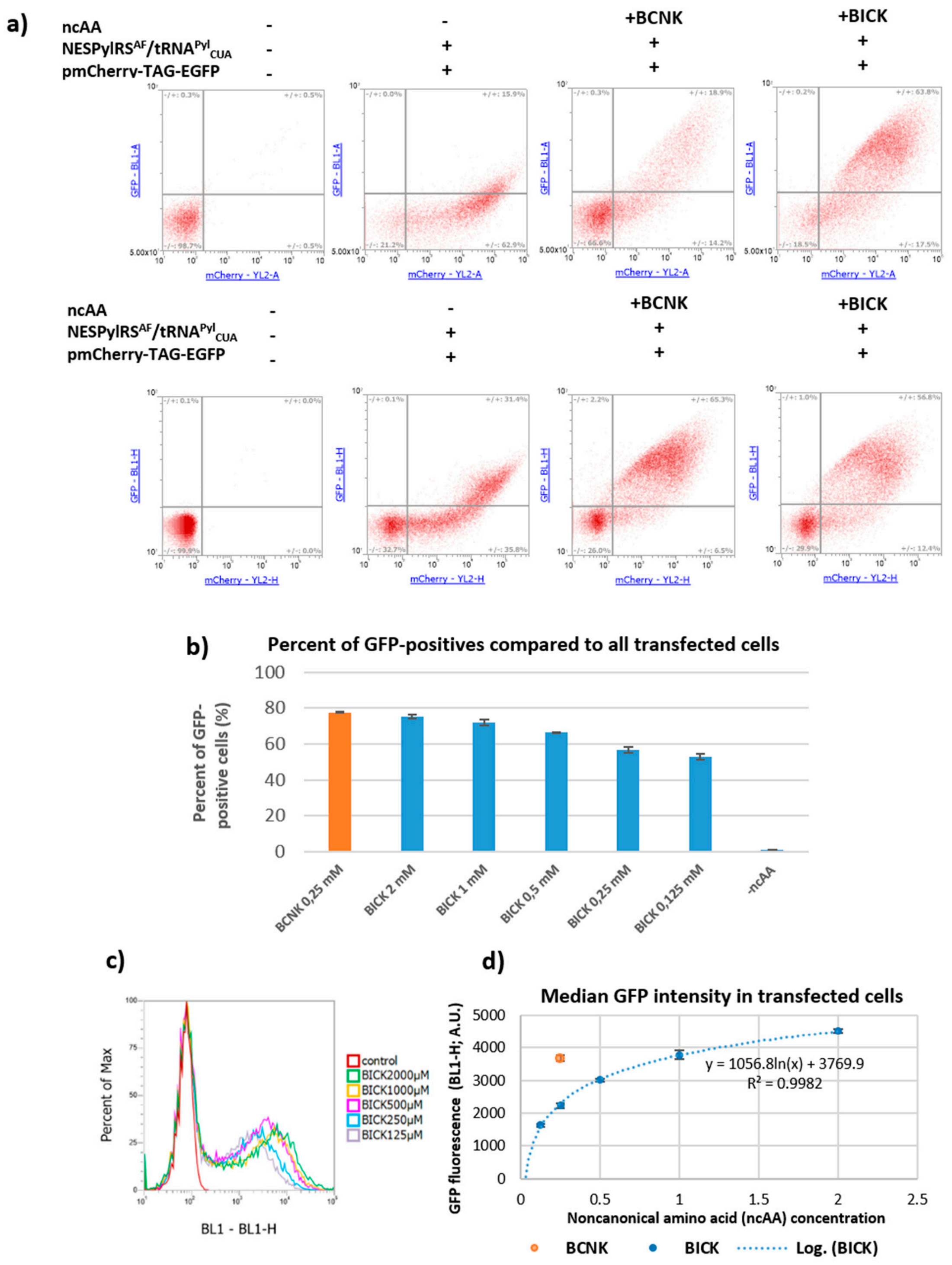

Figure S2.

Flow cytometry analysis of the reporter pmCherry-TAG-EGFP in HEK293T cells to assess the efficiency of genetic incorporation of **BICK** as compared to **BCNK**, or no ncAA added by NESPylRS^{AF}.

(a) Incorporation of 1 mM **BICK** as compared to 250 µM **BCNK**, or no ncAA added by NESPylRS^{AF}. On the horizontal axis fluorescence intensity corresponding to mCherry is shown, while GFP fluorescence is plotted on the vertical axis. The former refers to successfully transfected cells, while the latter indicates successful UAA incorporation and Amber suppression rates. Fluorescence intensity is indicated in arbitrary units. Upper and lower rows are results from independent experiments.

(b-d) Concentration dependent efficiency of BICK incorporation indicated by Amber stop codon correlated GFP fluorescence. Only those cells were considered GFP positive that had fluorescence intensities above GFP fluorescence in the no-UAA controls. **(b)** The percentage of GFP positive cells out of all mCherry positive cells was indicated. **(c)** Median fluorescence intensities of the GFP positive cell population were plotted against the applied BICK concentration values. A logarithmic correlation was found as indicated by the regression line fitted with R^2 value as high as 0.9982. **(d)** Representative histograms of flow cytometry analyses for the HEK293T cells receiving different concentrations of BICK. Error bars indicate SD (n = 3).

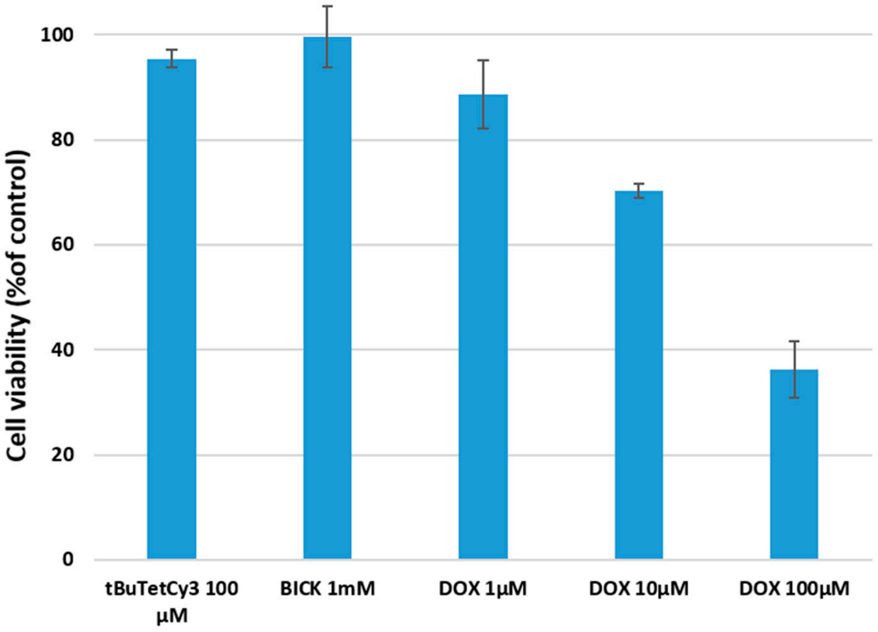

Figure S3.

MTT cell viability test of **BICK** and **tBuTetCy3** (**17**). The effect of 1 mM **BICK** and 100 µM **tBuTetCy3** on the viability of HEK293T cells after overnight (16 h) co-incubation was undetectable (**BICK**) or very slight (**tBuTetCy3**). Abbreviations: **tBuTetCy3**: Sulfo-Cy3-Tertbutyl-Tetrazine, **BICK**: isonitrile-carbamate-lysine, DOX: doxorubicin hydrochloride, positive control for cell toxicity. Error bars indicate SD (n = 3).

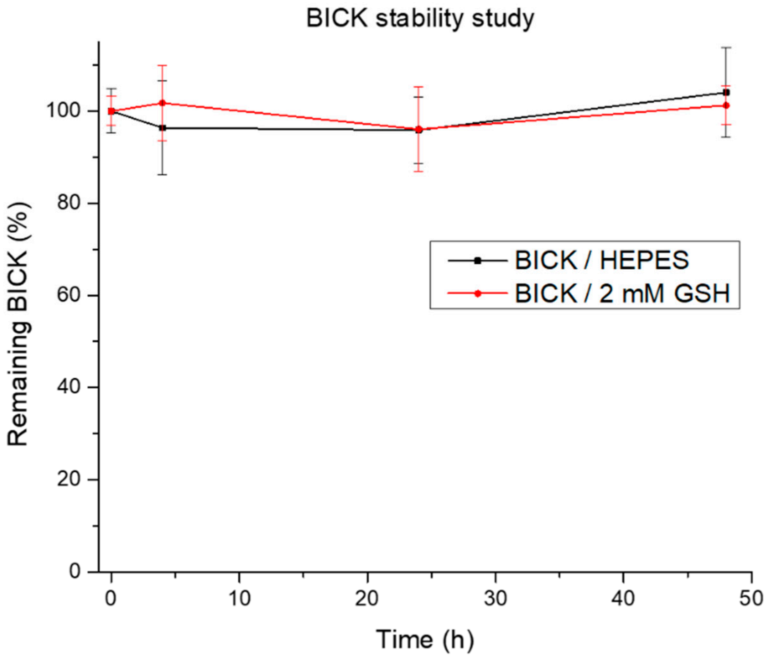

Figure S4.

Stability test of **BICK.** 1 mM **BICK** was incubated at 37°C for 48 hours in 20 mM HEPES buffer, pH 7.4, with or without the presence 2 mM reduced glutathione (GSH). Samples were taken at the indicated time points and the amount of remaining BICK was measured on a HPLC-ESI-MS system, using the inert HEPES as an internal standard. Error bars indicate SD (n = 3).

2. Supplementary Materials and Methods

2.1. Organic syntheses

2.1.1. General protocols

All starting materials were obtained from commercial suppliers (Sigma-Aldrich, Fluka, Merck, Alfa Aesar, Reanal, Molar Chemicals, Fluorochem) and used without further purification. (1R,8S,9s)-Bicyclo[6.1.0]non-4-yn-9-ylmethanol (BCN) and the corresponding succinimidyl carbonate (BCN-NHS) was obtained from Sigma. Analytical thin-layer chromatography (TLC) was performed on silica gel 60 F254 precoated aluminum TLC plates from Merck. Flash column chromatography was performed on Teledyne Isco CombiFlash® Rf+ automated flash chromatographer with silica gel (25-40 µm) from Zeochem. NMR spectra were recorded on a Varian Inova 500 MHz spectrometer. Chemical shifts (δ) are given in parts per million (ppm) using solvent signals or TMS as the reference. Coupling constants (J) are reported in Hertz (Hz). Analytical RP-HPLC-UV/Vis-MS measurements were employed using a Shimadzu LCMS-2020 instrument applying a Gemini C18 column (100 x 2.00 mm I.D.) in which the stationary phase is 5 µm silica with a pore size of 110 Å. The chromatograms were detected by a UV-Vis diode array (190-800 nm) and an ESI-MS detector. Linear gradient elution (0 min 0% B; 2.0 min 100% B; 3.5 min 100% B; 4.5 min 0% B; 5.0 min 0% B) with eluents A (2% HCOOH, 5% MeCN, and 93% H_2O) and B (2% HCOOH, 80% MeCN, and 18% H_2O) was used at a flow rate of 1.0 mL

min^{-1} at 30°C. The samples were dissolved in MeCN - H_2O mixture. Spectroscopic measurements were performed on a Jasco FP 8300 spectrofluorometer. Quartz cuvettes with path length of 1 cm were used. The exact masses were determined with an Agilent 6230 time-of-flight mass spectrometer.

2.1.2. Synthesis of BICK (4)

2-isocyano-2-methylpropyl (4-nitrophenyl) carbonate (2)

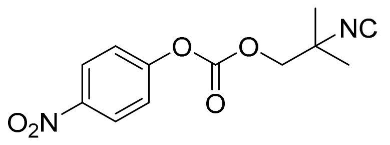

In a round-bottomed flask under nitrogen atmosphere 4,4-dimethyl-2-oxazoline (**1**, 532 µL; 5.04 mmol) was dissolved in anhydrous THF (20 mL), and cooled down to -78 °C. *n*-BuLi (2.15 mL; 5.04 mmol; 1.6 M in hexane) was added slowly to this solution and stirred for 1 h. *p*-Nitrophenyl-chloroformate (1016 mg; 5.04 mmol) was dissolved in 10 mL cold anhydrous THF, added to the reaction mixture, and stirred for another 30 min. After evaporation of the solvent, the crude product was dissolved in EtOAc and washed with 2 × 80 mL water, 80 mL brine, and dried over $MgSO_4$. After filtration the solvent was evaporated, and **2**, as an off-white, solid product (887 mg; 67%) was used without further purification. ^{1}H NMR (500 MHz, $CDCl_3$) δ 8.34 (d, *J* = 9.2 Hz, 2H), 7.50 (d, *J* = 9.2 Hz, 2H), 4.15 (s, 2H), 1.49 (s, 6H).

***N*^{2}-(((9*H*-fluoren-9-yl)methoxy)carbonyl)-*N*^{6}-((2-isocyano-2-methylpropoxy)carbonyl)lysine (3)**

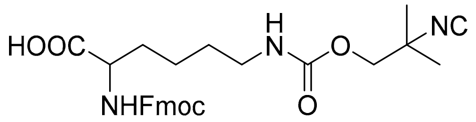

In a round-bottomed flask compound **2** (392 mg; 1.49 mmol), Fmoc-Lys-OH (500 mg; 1.24 mmol) and DIPEA (776 µL; 4.46 mmol) was dissolved in 2 mL anhydrous DMF. The reaction mixture was stirred for 30 min at room temperature. After evaporation of the solvent, the crude product was purified by column chromatography using CH_2Cl_2 / MeOH = 100/1 – 9/1 v/v to EtOAc / MeOH = 20/1 v/v gradient elution to furnish **3** as a pale yellow solid (436 mg; 94%). R_f = 0.32 (CH_2Cl_2 / MeOH = 9/1). LC-MS (ESI): m/z calcd for $C_{27}H_{30}N_3O_6^-$ [M-H]$^-$: 492; found 492.

***N*^{6}-((2-isocyano-2-methylpropoxy)carbonyl)lysine) (BICK, 4)**

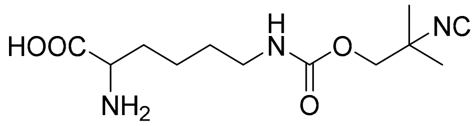

Compound **3** (436 mg; 1.13 mmol,) was dissolved in 5 mL CH_2Cl_2 and 2 mL piperidine was added. The reaction mixture was stirred for 20 min at room temperature, and the solvent was evaporated. After addition of Et_2O, a white precipitate formed, which was filtered and washed with methyl-*tert*-butyl-ether to yield **4** as a white solid (62 mg; 15%). ^{1}H NMR (500 MHz, CD_3OD) δ 4.11 (s, 2H), 3,59 (m, 1H), 3.21 (t, *J* = 7.0 Hz, 2H), 2.00 – 1,83 (m, 2H), 1.62 (m, 2H), 1.57 – 1.45 (m, 8H). ^{13}C NMR (126 MHz, CD_3OD) δ 172.9, 156.8, 153.8, 69.4, 57.0 (t, J=5.5 Hz), 54.7, 40.0, 30.6, 29.1, 24.6, 22.1. HRMS (ESI): m/z calcd for $C_{12}H_{22}N_3O_4^+$ [M+H$^+$]$^+$: 272.1605; found 272.1605.

2.1.3. Synthesis of 6-(6-(tert-butyl)-1,2,4,5-tetrazin-3-yl) nicotinic acid (7) and tBuTetRhod (9)

Ethyl pivalimidate (6)

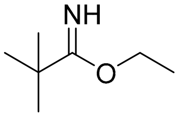

Pivalonitrile (**5**, 500 mg; 6.00 mmol) was dissolved in 5 mL EtOH, and 5 mL acetyl chloride was added dropwise. The reaction mixture was placed into the fridge overnight. The EtOH was removed by co-evaporation with hexane. After addition of Et_2O a white precipitate formed, which was filtered and dried *in vacuo* to give **6** as a white solid (992 mg; 63%). LC-MS (ESI): m/z calcd for $C_7H_{16}NO^+$ $[M+H]^+$: 130; found 130.

6-(6-(tert-butyl)-1,2,4,5-tetrazin-3-yl) nicotinic acid (7)

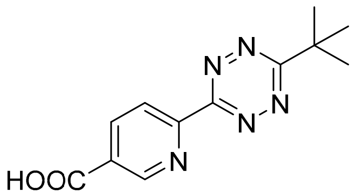

6-Cyanonicotinic acid (30 mg; 0.20 mmol), compound **6** (129 mg; 1.00 mmol), sulfur (5 mg; 0.14 mmol) and hydrazine monohydrate (212 µL, 6.60 mmol) were placed in a round-bottomed flask. The reaction mixture was stirred vigorously at 90 °C for 2 h. After cooling to room temperature, 50 mL 10% (m/m) citric acid solution was added to the mixture and extracted with EtOAc (4 × 50 mL). The combined organic phase was washed with brine (100 mL) and dried over $MgSO_4$. The solution was filtered, and the solvent was evaporated. The crude orange dihydrotetrazine (53 mg) was suspended in CH_2Cl_2 (5 mL), (diacetoxyiodo)benzene (97 mg; 0.30 mmol) was added and stirred overnight at room temperature. After evaporation of the solvent, the crude product was purified by flash chromatography (CH_2Cl_2 / MeOH = 100/1 – 9/1 v/v gradient elution) to yield **7** as a purple solid (44 mg; 84%). R_f = 0.50 (CH_2Cl_2 / MeOH = 2/1). ^1H NMR (500 MHz, $CDCl_3$) δ 9.60 (s, 1H), 8.76 (d, *J* = 8.2 Hz, 1H), 8.66 (dd, *J* = 8.2, 1.3 Hz, 1H), 1.47 (s, 9H). ^{13}C NMR (126 MHz, $CDCl_3$) δ 180.3, 176.7, 167.9, 162.8, 153.9, 152.3, 139.4, 128.4, 123.5, 38.4, 31.3. LC-MS (ESI): m/z calcd for $C_{12}H_{12}N_5O_2^-$ $[M-H]^-$: 258; found 258.

tBuTetRhod (9) (= *N*-(9-(2-(4-(6-(6-(tert-butyl)-1,2,4,5-tetrazin-3-yl)nicotinoyl)piperazine-1-carbonyl)phenyl)-6-(diethylamino)-3*H*-xanthen-3-ylidene)-*N*-ethylethanaminium chloride)

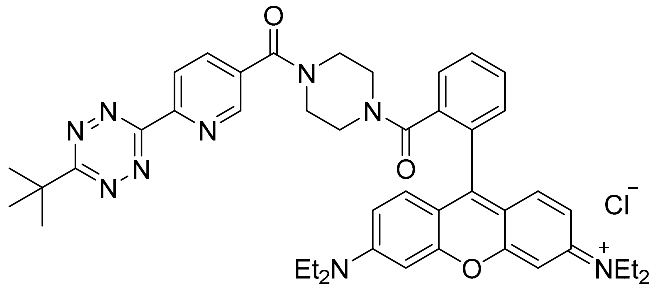

In a round-bottomed flask, compound **7** (21 mg; 0.08 mmol) was dissolved in 6 mL dry MeCN, HBTU (29 mg; 0.08 mmol), HOBt × H_2O-t (13 mg; 0.08 mmol), DIPEA (42 µL; 0.24 mmol) were added and the rhodamine-piperazine derivative (**8**) (59 mg; 0.11 mmol) after 5 minutes. The reaction mixture was stirred for 4 h at room temperature and then the solvent was evaporated. The residue was purified by flash chromatography using CH_2Cl_2 / MeOH = 100/1 – 9/1 as eluent to furnish **9** as a dark purple solid (17 mg; 29 %). R_f = 0.50 (CH_2Cl_2 / MeOH = 9/1). ^1H NMR (500 MHz, $CDCl_3$) δ 8.89 (d, *J* = 1.5 Hz, 1H), 8.71 (d, *J* = 8.1 Hz, 1H), 8.07 (dd, *J* = 8.1, 2.2 Hz, 1H), 7.67 (s, 2H), 7.55 (s, 1H), 7.32 (s, 1H), 7.25 (s, 1H), 7.23 (s, 1H), 6.99 (s, 2H), 6.75 (s, 2H), 3.72 – 3.55 (m, 16H), 1.64 (s, 9H), 1.33 (t, *J* = 7.1 Hz, 12H). HRMS (ESI): m/z calcd. for $C_{44}H_{50}O_3Na^+$ $[M+Na]^+$: 752.4037; found 752.4030.

2.1.4. Synthesis of Sulfo-Cy3 carboxylic acid (14)

1-(5-carboxypentyl)-2,3,3-trimethyl-3H-indol-1-ium-5-sulfonate (11)

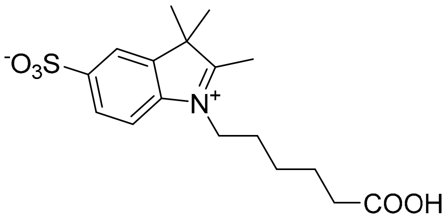

Potassium 2,3,3-trimethyl-3H-indole-5-sulfonate (**10**, 1.00 g, 3.6 mmol) and 6-bromohexanoic acid (2.10 g, 10.8 mmol) were suspended in dichlorobenzene (10 mL). KI (60 mg, 0.36 mmol) was added and the reaction mixture was stirred under nitrogen at 150 °C for 48 hours. After completion of the reaction, the reaction mixture was cooled to room temperature, then at 4 °C for 1 hour. The precipitate was collected by filtration and washed with dichlorobenzene, ^{i}PrOH:Et_2O (1:1 v/v) and finally with Et_2O to yield 510 mg (40%) violet powder. ^{1}H NMR (500 MHz, DMSO-d_6) δ 8.01 (s, 1H), 7.93 (d, *J* = 7.6 Hz, 1H), 7.82 (d, *J* = 7.6 Hz, 1H), 4.45 (s, 2H), 3.56 (m, 2H), 2.85 (s, 3H), 2.22 (m, 2H), 1.84 (m, 2H), 1.54 (s, 6H), 1.42 (s, 2H). ^{13}C NMR (126 MHz, DMSO-d_6) δ 174.2, 151.5, 149.5, 141.5, 126.3, 120.7, 118.5, 114.9, 54.2, 47.6, 33.3, 26.9, 25.4, 24.0, 21.9, 14.1. LC-MS (ESI): m/z calcd for $C_{17}H_{22}NO_5S^-$ [M-H]$^-$: 352; found 352.

1,3,3-trimethyl-2-(2-(N-phenylacetamido)vinyl)-3H-indol-1-ium-5-sulfonate (13)

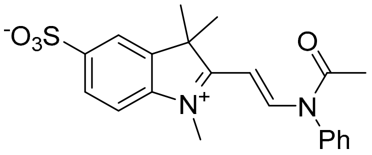

1,2,3,3-tetramethyl-3*H*-indol-1-ium-5-sulfonate (**12**, 254 mg, 1.0 mmol) and *N,N'*-diphenylformamidine (393 mg, 2.0 mmol) were dissolved in acetic acid (1.4 mL) and acetic anhydride (1.4 mL) under nitrogen. The reaction mixture was stirred at 120 °C for 1 hour, then allowed to cool to room temperature. EtOAc (8 mL) was added and the resulting precipitate was filtered, washed with EtOAc, dried *in vacuo*, and used immediately without further purification.

Sulfo-Cy3 carboxylic acid (14) (= 2-(3-(1-(5-carboxypentyl)-3,3-dimethyl-5-sulfoindolin-2-ylidene)prop-1-en-1-yl)-1,3,3-trimethyl-3H-indol-1-ium-5-sulfonate)

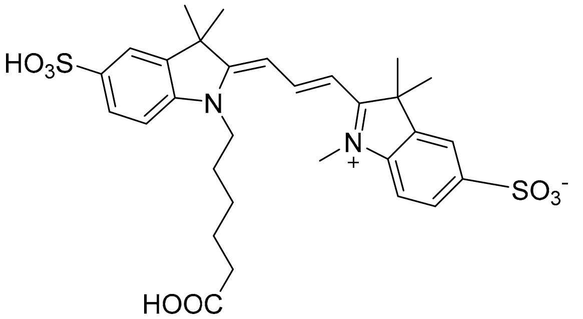

The freshly prepared 1,3,3-trimethyl-2-(2-(*N*-phenylacetamido)vinyl)-3*H*-indol-1-ium-5-sulfonate (**13**, 1.0 mmol) and 1-(5-carboxypentyl)-2,3,3-trimethyl-3*H*-indol-1-ium-5-sulfonate (**11**, 177 mg, 0.5 mmol) were dissolved in pyridine (3 mL) and acetic anhydride (0.3 mL) under nitrogen. The reaction mixture was stirred at room temperature for 16 hours. Addition EtOAc (10 mL) resulted in a gummy precipitate, which was purified by flash column chromatography (1% HCOOH in 0% -> 30% MeOH in DCM gradient elution). Evaporation of the solvent yielded 49 mg (16% for 2 steps) of the desired dye as a sticky powder. ^{1}H NMR (500 MHz, CD_3OD) δ 8.57 (t, *J* = 13.4 Hz, 1H), 7.95 (s, 2H), 7.90 (d, *J* = 8.3 Hz, 2H), 7.39 (d, *J* = 8.2 Hz, 2H), 6.51 (dd, *J* = 13.5, 4.3 Hz, 2H), 4.18 (t, *J* = 7.0 Hz, 2H), 3.71 (s, 3H), 2.32 (t, *J* = 7.2 Hz, 2H), 1.90 – 1.82 (m, 2H), 1.79 (s, 12H), 1.72 – 1.67 (m, 2H), 1.55 – 1.48 (m, 2H). ^{13}C NMR (126 MHz, CD_3OD)

δ 177.6, 176.8, 152.8, 145.4, 144.7, 142.2, 142.1, 141.3, 128.3, 128.2, 128.14, 128.08, 122.9, 121.5, 121.4, 112.1, 112.0, 104.9, 104.7, 77.7, 50.7, 45.3, 34.5, 32.1, 28.2, 28.1, 27.2, 25.6. LC-MS (ESI): m/z calcd for $C_{30}H_{37}N_2O_8S_2^+$ $[M+H]^+$: 617; found 617.

2.1.5. Synthesis of tBuTetCy3 (17)

Sulfo-Cy3 NHS ester (15)

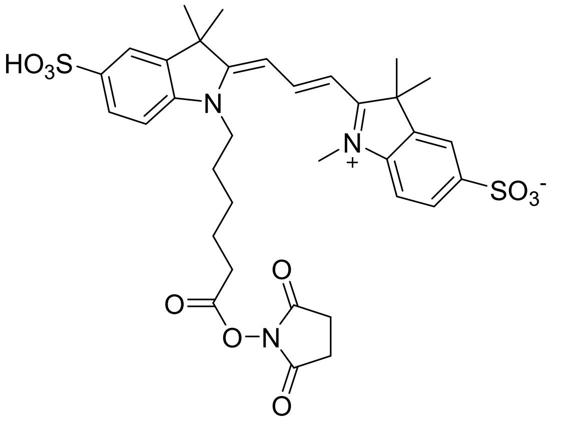

Sulfo-Cy3 (**14**, 30 mg, 0.05 mmol) and *N*-hydroxysuccinimide (12 mg, 0.10 mmol) was dissolved in DCM (5 ml). EDC×HCl (20 mg, 0.10 mmol) was added, and the reaction mixture was stirred at 25 °C for 2 hours. After LC-MS indicated full conversion, the solvent was evaporated, and the crude product was immediately used in the next step without purification. LC-MS (ESI): m/z calcd for $C_{34}H_{40}N_3O_{10}S_2^+$ $[M+H]^+$: 714; found 714.

Sulfo-Cy3 amine (16)

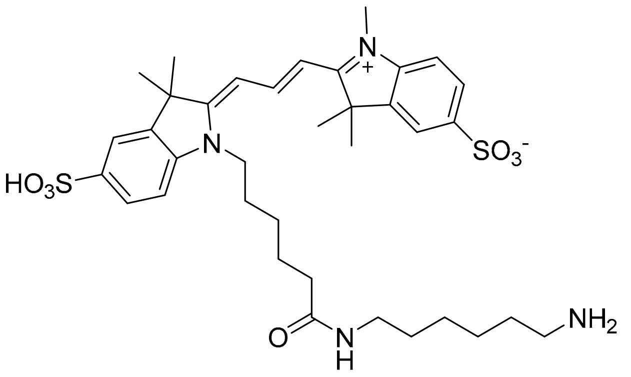

The crude sulfo-Cy3 NHS ester (**15**, 0.05 mmol) was dissolved in dry DMF (5 ml), then hexane-1,6-diamine (12 mg, 0.10 mmol) was added, and the reaction mixture was stirred at 25 °C for 16 hours. After evaporation of the solvent, the crude product was purified by RP-HPLC (0.1% HCOOH in H_2O -> 0.1% HCOOH in MeCN gradient elution) to yield 15 mg (42%, 2 steps) of the desired product, which still contained water after lyophilization, and was used wet in the next step. LC-MS (ESI): m/z calcd for $C_{36}H_{51}N_4O_7S_2^+$ $[M+H]^+$: 715; found 715.

Sulfo-Cy3 – ^{t}butil-tetrazine (tBuTetCy3, 17)

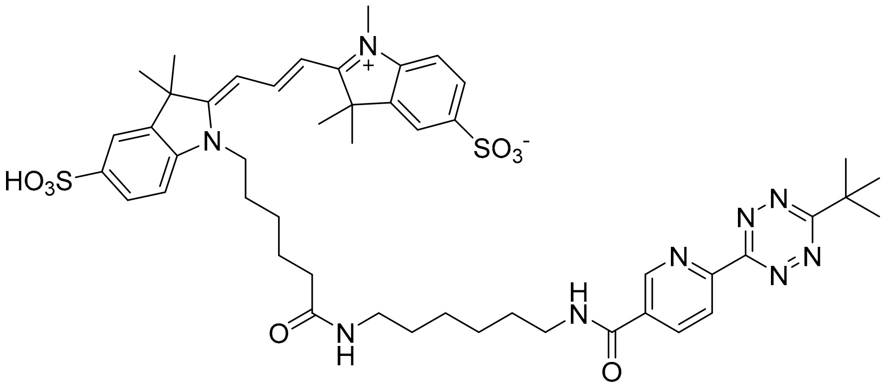

6-(6-(tert-butyl)-1,2,4,5-tetrazine-3-yl) nicotinic acid (**7**, 15 mg, 0.06 mmol), HBtU (21 mg, 0.056 mmol) and HOBt×H_2O (10 mg, 0.06 mmol) were mixed and dissolved in anhydrous acetonitrile (4 mL) and stirred under nitrogen atmosphere for 10 minutes at 25 °C. To the resulting solution EDIPA (17 µL, 0.1 mmol), then a solution Sulfo-Cy3 amine (**16**, 14 mg, 0.02 mmol) in anhydrous acetonitrile (2 mL) were added. The reaction mixture was stirred for 4 hours at 25 °C. The solvent was removed on a rotary evaporator and the crude product was purified by flash column chromatography (0% -> 20% MeOH in DCM gradient elution) twice to yield 5 mg (26%) dye. ^{1}H NMR (500 MHz, CD_3OD) δ 9.36 (s, 2H), 8.71 (m, 3H), 8.59 (m 3H), 7.93 – 7.89 (m, 4H), 6.49 (dd, *J* = 13.3, 6.5 Hz, 2H), 4.17 (t, *J* = 7.3 Hz, 2H), 3.71 (s, 3H), 3.17 – 3.12 (m, 6H), 2.38 – 2.31 (m, 2H), 2.20 (t, *J* = 7.1 Hz, 2H), 2.08 – 2.02 (m, 2H), 1.88 – 1.84 (m, 2H), 1.79 (s, 12H), 1.64 (s, 9H), 1.51 – 1.46 (m, 6H). HRMS (ESI): m/z calcd. for $C_{48}H_{60}N_9O_8S_2^-$ [M-H]$^-$: 954.4011; found 954.4017.

2.1.6. Synthesis of BCN-HaloTag substrate (21)

***tert*-butyl (2-(2-((6-chlorohexyl)oxy)ethoxy)ethyl)carbamate (19)**

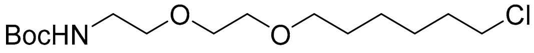

N-Boc-2-(2-hydroxy-ethoxy)-ethylamine (**18**, 2.00 g, 9.75 mmol) was dissolved in THF (14 mL) and DMF (7 mL) at 0 °C, NaH (470 mg, 60% in mineral oil, 11.7 mmol) was added to the solution, and stirred for 30 min at 0 °C. Then 1-chloro-6-iodohexane (2 mL, 13.65 mmol) was added, and stirred overnight. After quenching with saturated NH_4Cl the reaction mixture was extracted with EtOAc (3 × 30 mL), washed with H_2O and brine, then dried over Na_2SO_4 and evaporated. The crude product was purified by silica gel column chromatography using EtOAc/hexane (20/80 to 30/70, v/v) to yield the pure product (2.10 g, 67%) as colourless oil. ^{1}H NMR (500 MHz, $CDCl_3$) δ 5.00 (s, 1H), 3.61 – 3.57 (m, 2H), 3.56 – 3.49 (m, 6H), 3.45 (t, *J* = 6.6 Hz, 2H), 3.30 (m, 2H), 1.80 – 1.73 (m, 2H), 1.63 – 1.54 (m, 2H), 1.49 – 1.43 (m, 2H), 1.43 (s, 9H), 1.40 – 1.33 (m, 2H). ^{13}C NMR (126 MHz, $CDCl_3$) δ 156.1, 79.3, 71.4, 70.4, 70.3, 70.2, 45.1, 40.5, 32.7, 29.6, 28.6, 26.8, 25.5. LC-MS (ESI): m/z calcd for $C_{15}H_{31}ClNO_4^+$ [M+H]$^+$: 324; found 324.

2-(2-((6-chlorohexyl)oxy)ethoxy)ethan-1-amine (20)

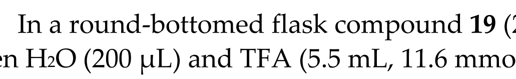

In a round-bottomed flask compound **19** (2.00 g, 6.18 mmol) was dissolved in anhydrous CH_2Cl_2 (16 mL) at 0 °C, then H_2O (200 µL) and TFA (5.5 mL, 11.6 mmol) was added. The reaction mixture was stirred for 2 hours at room temperature. After evaporation of the solvent the crude product was treated with anhydrous K_2CO_3 (9.80 g, 11.5 mmol) in MeOH (33 mL). The reaction mixture was filtered, and the filtrate was concentrated. The residue was then purified by column chromatography (impurities: EtOAc/hexane = 60/40, v/v, product: 2-5% methanol in CH_2Cl_2) to get compound **14** as a colorless oil (1.20 g, 90%). ^{1}H NMR (500 MHz, $CDCl_3$) δ 3.70 – 3.65 (m, 2H), 3.65 – 3.61 (m, 2H), 3.56 (m, 2H), 3.52 (t, *J* = 6.7 Hz, 2H), 3.45 (t, *J* = 6.7 Hz, 2H), 3.06 (s, 2H), 1.97 (s, 3H), 1.82 – 1.71 (m, 2H), 1.62 – 1.55 (m, 2H), 1.50 – 1.40 (m, 2H), 1.39 – 1.31 (m, 2H). ^{13}C NMR (126 MHz, $CDCl_3$) δ 71.4, 70.5, 70.0, 68.4, 45.1, 39.9, 32.7, 29.5, 26.8, 25.5. LC-MS (ESI): m/z calcd for $C_{10}H_{23}ClNO_2^+$ [M+H]$^+$: 224; found 224.

((1R,8S,9s)-bicyclo[6.1.0]non-4-yn-9-yl)methyl (2-(2-((6-chlorohexyl)oxy)ethoxy)-ethyl)carbamate (21)

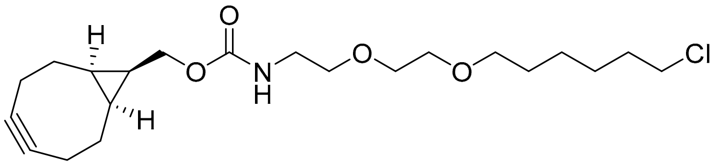

A solution of compound **20** (85 mg, 0.38 mmol), BCN-NHS (55 mg, 0.19 mmol) and diisopropylethylamine (100 µL, 0.57 mmol) in acetonitrile (3 mL) was stirred at room temperature for 16 hours. After evaporation of the solvent the residue was dissolved in EtOAc (15 mL) and washed with 10% w/w K_2CO_3 solution, dried over Na_2SO_4 and concentrated. The crude product was purified by flash column chromatography (hexane/EtOAc = 5/1) to yield compound **21** as a pale-yellow oil (70 mg, 92%). R_f = 0.26 (hexane/EtOAc = 3/1). ^{1}H NMR (500 MHz, $CDCl_3$) δ 5.17 (s, 1H), 4.13 (d, *J* = 7.9 Hz, 2H), 3.61 – 3.57 (m, 2H), 3.56 – 3.53 (m, 4H), 3.51 (t, *J* = 6.7 Hz, 2H), 3.45 (t, *J* = 6.6 Hz, 2H), 3.38 – 3.33 (m, 2H), 2.31 – 2.16 (m, 6H), 1.80 – 1.73 (m, 2H), 1.63 – 1.54 (m, 4H), 1.47 – 1.41 (m, 2H), 1.40 – 1.30 (m, 3H), 1.01 – 0.87 (m, 2H). ^{13}C NMR (126 MHz, $CDCl_3$) δ 156.9, 98.9, 71.4, 70.5, 70.22, 70.17, 62.8, 45.1, 40.9, 32.7, 29.6, 29.2, 26.8, 25.5, 21.5, 20.3, 17.9. LC-MS (ESI): m/z calcd for $C_{21}H_{35}ClNO_4^+$ [M+H]$^+$: 400; found 400.

2.1.7. Synthesis of NHS-NC (23)

2,5-dioxopyrrolidin-1-yl (2-isocyano-2-methylpropyl) carbonate (23, NHS-NC)

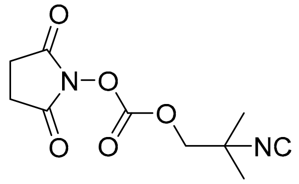

n-BuLi (2.5 M in hexanes; 2 mL; 21.2 mmol) was added slowly to a solution of 4,4-dimethyl-2-oxazoline (530 µL; 5 mmol) in dry THF (5 mL) at -78 °C. The solution was stirred for 1 h at -78 °C, then quickly transferred to a stirred solution of *N*,*N*′-disuccinimidyl carbonate (3,84 g; 15 mmol) in dry MeCN (15 mL) that had been pre-cooled to -78 °C. After 30 minutes the solution was allowed to reach 25 °C, concentrated onto cellite and purified by flash column chromatography using hexanes / EtOAc as eluent. Removal of the solvent yielded **23** as a colourless oil (335 mg; 28%). R_f = 0.83 (EtOAc / MeOH : 9/1). ^{1}H NMR (500 MHz, $CDCl_3$) δ 4.25 (s, 1H), 2.85 (s, 2H), 1.51 (s, 3H). ^{13}C NMR (126 MHz, $CDCl_3$) δ 168.4, 157.4, 151.4, 75.1, 56.2, 25.8, 25.6. HRMS (ESI): m/z calcd. for $C_{10}H_{12}N_2O_5Na^+$ [M+Na]$^+$: 263.0644; found 263.0645.

2.1.8. Other compounds

***N*-(6-(diethylamino)-9-(2-(piperazine-1-carbonyl)phenyl)-3*H*-xanthen-3-ylidene)-*N*-ethylethanaminium chloride (8, Rhodamine-piperazine)**

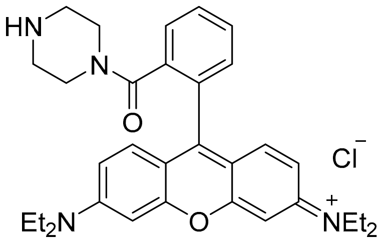

The rhodamine-piperazine derivative (**8**) was prepared earlier as described in the literature [28].

3-(4-bromophenyl)-6-methyl-1,2,4,5-tetrazine (22)

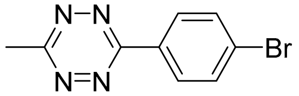

The **22** tetrazine derivative was prepared earlier as described in the literature [20].

((E)-3,7-bis(dimethylamino)-5,5-dimethyl-6'-(2-(6-methyl-1,2,4,5-tetrazin-3-yl)vinyl)-3'H,5H-spiro[dibenzo[b,e]siline-10,1'-isobenzofuran]-3'-one (24, Tet-SiR)

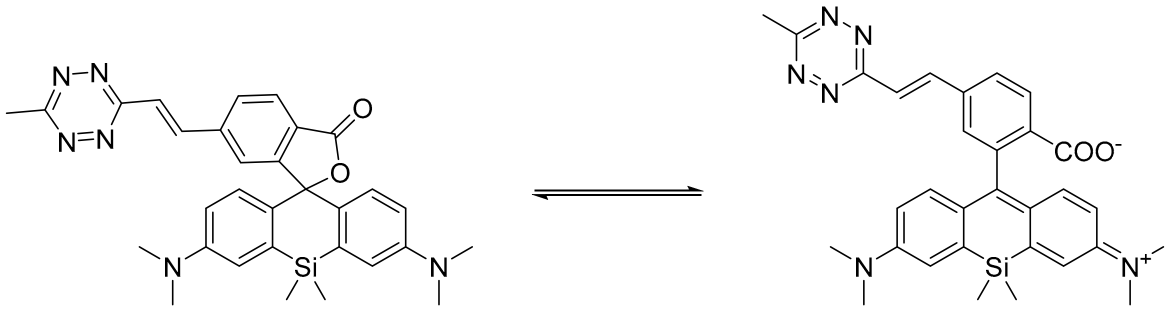

Tet-SiR (**24**) was prepared earlier as described in the literature [20].

(1-Ethyl-2-((*E*)-3-((*E*)-1-ethyl-3,3-dimethyl-5-((*E*)-2-(6-methyl-1,2,4,5-tetrazin-3-yl)vinyl)indolin-2-ylidene)prop-1-en-1-yl)-3,3-dimethyl-3*H*-indol-1-ium-5-sulfonate) (25, TetCy3)

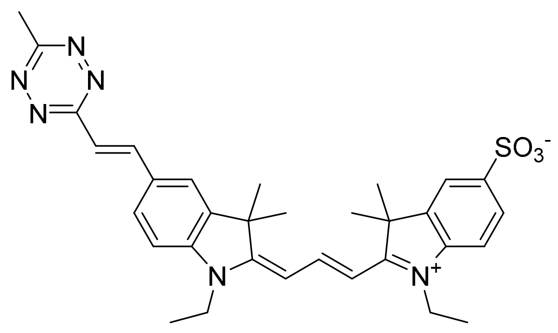

TetCy3 (**25**) was prepared earlier as described in the literature [17].

2.2. BICK amino acid stability testing

Stability of the BICK amino acid was studied in HEPES buffer with or without the presence of GSH. 100 mM stock solution of BICK was freshly prepared in 20 mM HEPES buffer, pH 7.4. A 20 mM stock solution of GSH was prepared likewise. The stock solutions were diluted in the 20 mM HEPES buffer, pH 7.4 to give a solution of 1 mM BICK or 1 mM BICK combined with 2 mM GSH. The mixtures were incubated at 37 °C for 48 hours, during which 80 µl samples were taken at the indicated time points; diluted 10 times, and the amount of remaining BICK was measured on a HPLC-ESI-MS system, using the inert HEPES as an internal standard. Samples were prepared and measured in three replicates.

2.3. Activation and conjugation of proteins for in-gel fluorescence studies, and SDS-PAGE

2.3.1.Conjugation of model proteins

Bovine serum albumin (BSA, 70 µM) or Transferrin (TF, 70 µM) and 350 µM activated isonitrile derivative **23** or BCN-NHS (from the 20 mM stock solution in DMSO) were mixed and incubated for 60 minutes at room temperature in 110 mM Na_2CO_3/$NaHCO_3$ buffer (pH 9.0). The excess reagents were removed by using a SpinPrep column (Sigma, St Louis, MO, USA) filled with Sephadex G-25 "Fine" desalting gel (Pharmacia Fine Chemicals, Sweden). This procedure resulted a buffer exchange also to PBS (pH 7.4). In the next step, **tBuTetRhod** (500 µM), and/or **Tet-SiR** (500 µM) was added for 2 hours. The excess reagents were removed again by using a SpinPrep column filled with Sephadex G-25 "Fine" desalting gel. The labeled proteins were separated by SDS-PAGE.

2.3.2. SDS polyacrylamide gel electrophoresis

The samples were diluted with a sample buffer (250 mM Tris-HCl (pH 6.8) + 10% SDS + 40% glycerol + 0.02% bromophenol blue + 400 mM DTT) in a 3/1 ratio. The size of the polyacrylamide gels was 8.5 cm × 7.5 cm × 0.1 cm. They were the combination of 4% concentration and 8% separation PAGE gels (acrylamide/bisacrylamide ratio was 29/1; and concentration gel buffer was 125 mM Tris-HCl + 0.1% SDS (pH 6.8) and the separation gel buffer was 375 mM Tris-HCl + 0.1% SDS (pH 8.8)). PageRuler Plus Prestained Protein Ladder (Thermo Fisher Scientific) was applied as the molecular weight standard. Separations were carried out in a Mini-Protean Tetra Cell (Bio-Rad, Hercules, CA, USA) using 25 mM Tris/192 mM glycine + 0.1% SDS (pH 8.3) as the running buffer and 150 V voltage for 70 min at 25 °C. The gels were documented by using a Biorad Bio-Rad ChemiDoc™ Imager. **tBuTetRhod** was detected in the Cy3 channel (602/50 nm) and **Tet-SiR** in the Cy5 channel (700/50 nm). Furthermore, the gels were stained for proteins with Coomassie Brilliant-Blue.

2.4. Plasmids

All restriction endonucleases were purchased from NEB (New England Biolabs, MA, USA), when not stated otherwise.

2.4.1. Construction of pmCherry-TAG-EGFP-HA reporter plasmid

For flow cytometric evaluation of genetic incorporation of the novel ncAA BICK, we cloned the reporter unit mCherry-TAG-EGFP from the plasmid pPCKRS-mCherry-TAG-EGFP-HA, a (gift from Jason Chin's lab, Cambridge, UK) [14] by digestion with the restriction enzymes HpaI and NheI and ligating the resulting insert into the vector backbone generated from digestion of plasmid H2B-GFP, a gift from Geoff Wahl (Addgene plasmid # 11680 ; http://n2t.net/addgene:11680 ; RRID:Addgene_11680) [29] with the same enzymes.

2.4.2. Construction of HaloTag plasmids

Tomm20-HaloTag

The HaloTag fusion protein plasmid pHaloTag-EGFP was a gift from Thomas Leonard & Ivan Yudushkin (Addgene plasmid # 86629 ; http://n2t.net/addgene:86629; RRID:Addgene_86629) [30]. The HaloTag insert was generated by PCR amplification and cutting with BamHI on the 5′ end, and with NotI on the-3′ end. A stop codon (TAA) was added to the HaloTag sequence that now we fused to the C termini of proteins of interest. The fragment corresponding to mCherry was removed from the Tomm20-N-mCherry plasmid by cutting with also BamHI/NotI, then the HaloTag insert was ligated to gain the Tomm20-HaloTag plasmid. The Tomm20-N-mCherry plasmid was a kind gift from Michael Davidson (Addgene plasmid # 55146 ; http://n2t.net/addgene:55146 ; RRID:Addgene_55146).

Primer sequences:
HaloTag BamHI FW
5′-GTAGCGTGGATCCACCGGTCGCCACCATGGCAGAAATCGGTACT-3′
HaloTag NotI Rev
5'-GAGTCGCGGCCGCTTTAGCCGGAAATCTCGAGCG-3'

Vimentin-HaloTag

A plasmid containing Vimentin (pVimentin-miRFP703) was a gift from Vladislav Verkhusha (Addgene plasmid #79996; http://n2t.net/addgene:79996 ; RRID:Addgene_79996) [31]. The Vimentin ORF was cut by restriction endonucleases NheI and BamHI, and the resulting insert was used to replace the Tomm20 ORF in the Tomm20-Halotag plasmid.

H2B-HaloTag

The Tomm20 insert was removed from the Tomm20-HaloTag plasmid by cutting with NheI (NEB, #R3131) and BamHI (NEB, #R3136) restriction endonucleases to gain a vector backbone with compatible ends to the insert fragment coding for the H2B histon protein cleaved from the purchased H2B-GFP plasmid (Addgene, #11680) with the same enzymes. The vector and insert fragments were ligated to gain the plasmid H2B-HaloTag.

LaminA-HaloTag

The insert encoding the LaminA protein was created by PCR using the mCherry-LaminA-C-18 plasmid, a gift from Michael Davidson (Addgene plasmid # 55068 ; http://n2t.net/addgene:55068; RRID:Addgene_55068) as a template. The PCR product was cut with NheI and BamHI, and was ligated into the same HaloTag vector backbone as above.

Primer sequences:

Lamin NheI FW

5′-TTATTGCTAGCGCCACCATGGTAGAGACCCCGTCCCAGCG-3′

Lamin BamHI Rev

5′-CCCCAGAACTGCAGCATCATGGGGCGGTGGGGATCCATTAAT-3′

2.5. Cell culture

Human embryonic kidney HEK-293T cell line was purchased from American Type Culture Collection (ATCC; CRL-3216, Manassas, VA, USA) and african green monkey kidney fibroblast (COS-7) cell line was purchased

from Sigma-Aldrich (Sigma 87021302, St. Louis, MO, USA). HEK293T and COS-7 cells were both maintained in Dulbecco's modified Eagle's medium (Gibco 21063-029) supplemented with 1% penicillin-streptomycin (Gibco Life Technologies 15140-122), 1% L-GlutaMAX (Gibco 35050-061), 1% sodium pyruvate (Gibco 11360-070), and 10% FBS (Gibco 10500-064). Cells were cultured at 37°C in a 5% CO_2 atmosphere and passaged –using trypsin (Gibco 25200-056) every 3–4 days up to 20 passages.

For HEK293T cells 0.01 mg/ml Poly-L-lysine (Sigma P5899) was used to coat the chambers for 4-8h, then the cells were seeded at 160 000 cells/ml density 20-24 h prior to transfection, resulting in 70-80% confluency at the time of transfection. COS-7 cells were seeded without Poly-L-lysine coating at 40 000 cells/ml density 48 h prior to transfection. In vitro and in vivo labeling experiments were performed on cells seeded into µ-Slide 8 well plates (Ibidi 80827; 250 µl/well). Cells for flow cytometry experiments were seeded on 12-well plates (Greiner Bio-One 665 180; 1 ml/well). MTT tests were carried out in BioLite 48-well plates (Thermo Fisher Scientific, 130187; 250 µl/well).

2.6. Flow cytometry

HEK293T cells were seeded into 12-well plates (Greiner Bio-One 665 180; 1 ml/well) as described in 4.3. Cells were maintained at 37°C in a 5% CO_2 atmosphere during all incubation steps. Cells were transfected with Jetprime® transfection reagent (Polyplus 114-07) according to the protocol of the manufacturer. Transfections were performed with plasmids pmCherry-^{TAG}-EGFP (0.8 µg/well) and NESPylRS^{AF}/tRNA$^{Pyl}_{CUA}$ (1 µg/well). Medium was exchanged for fresh medium containing the ncAA 4h post-transfection and left until the time of harvesting. For flow cytometry, cells were harvested on the day after transfection, and trypsinized, and resuspended in DMEM. The cellular fluorescence of the cells was determined within an hour using an Attune NxT Flow Cytometer (Life Technologies, Carlsbad, CA, USA). The figures were generated using the Attune NxT Software v3.1.2. Cells were gated to single cells after excluding debris and aggregates. Fluorescence was acquired with a yellow-green laser excitation (561 nm) in the YL2 channel (620/15 nm) for mCherry and with a blue laser excitation (488 nm) in the BL1 channel (530/30) for GFP.

2.7. Bioorthogonal labeling of proteins in live HEK293T cells

HEK293T cells were seeded into µ-Slide 8 well plates (Ibidi 80827) as described in 4.2. Cells were maintained at 37°C in a 5% CO2 atmosphere during all incubation steps. Cells were transfected with Jetprime® transfection reagent (Polyplus 114-07) according to the protocol of the manufacturer.

For single dye labeling, plasmids IRK^{676TAG}-GFP and NESPylRS^{AF}/tRNA$^{Pyl}_{CUA}$ plasmids (gift from Dr. Edward Lemke's lab [9, 32]) were added to the Jetprime® mixture. After 4 hours of transfection, cell medium was exchanged to DMEM containing 1 mM BICK or 250 µM BCNK, and cells were incubated overnight.

On the next day, cells were washed once with DMEM, then cells in dedicated wells received 100 µM tBu-TetCy3 in DMEM, and were incubated with it overnight. The next day, other wells according to the experimental settings received 3 µM TetCy3 for 90 min. Dyes were then removed, cells were washed 3× quickly in indicator-free DMEM, left to wash for further 2 hours, then were imaged.

For double labeling, plasmids IR^{K676TAG}-GFP and NESPylRS^{AF}/tRNA$^{Pyl}_{CUA}$ plasmids (gift from Dr. Edward Lemke's lab [9,25]) along with either one of the HaloTag vectors H2B-HaloTag or LaminA-HaloTag were added at equal amounts of 0.13 µg each. After 4 hours of transfection, cell medium was exchanged to DMEM containing 1 mM BICK, and cells were incubated overnight.

On the next day cells were washed once with DMEM, then received 6 µM HaloTag-ligand BCN for 60 min. After washing quickly once with DMEM, first labeling with 10 µM **Tet-SiR** for 60 min followed. **Tet-SiR** was then removed, cells were washed 3× quickly with DMEM, then were washed for further 2 hours. The second dye, 100 µM

tBuTetCy3 was then added, and cells were incubated with it overnight. The next day, cells were washed 3× quickly in Indicator-free DMEM, then were imaged in the latter after 2 hours of washing.

2.8. Bioorthogonal labeling of proteins in fixed cells

2.8.1. Activation of a secondary antibody with NHS-isonitrile (23)

Secondary antibodies (16 μM goat anti-rabbit IgG, Jackson ImmunoResearch, 111-005- 003) was conjugated with 400 μM activated isonitrile derivative **NHS-NC** (**23**) and incubated for 30 minutes at room temperature in 110 mM Na_2CO_3/$NaHCO_3$ buffer (pH 9.0). The excess reagents were removed by using a SpinPrep column (Sigma, St Louis, MO, USA) filled with Sephadex G-25 "Fine" desalting gel (Pharmacia Fine Chemicals, Sweden). This procedure resulted a buffer change also to PBS (pH 7.4). For positive controls, **tBuTetRhod** was added in 600 μM concentration for 60 min in the next step. The excess reagents were removed again by using a SpinPrep column filled with Sephadex G-25 "Fine" desalting gel.

2.8.2. Dual staining of TOMM20 and Vimentin or LaminA after fixation.

COS-7 cells were seeded onto μ-Slide 8 well plates (Ibidi 80827) as described in section 4.2. Cells were maintained at 37°C in a 5% CO_2 atmosphere during all incubation steps. Cells were transfected with Lipofectamine™ 3000 Transfection Reagent (L3000008) according to the manufacturer's protocol. Either one of the HaloTag expression plasmids Vimentin-HaloTag or LaminA-HaloTag were added at 0.25 μg quantity each. After 4-6 hours of transfection, cell medium was exchanged to DMEM, and cells were incubated overnight.

On the next day cells were washed once with DMEM, then received 6 μM Halo -BCN for 90 min. Cells were then washed 3× quickly with DMEM, then were washed for further 2 hours. Afterwards, the samples were gently washed with pre-warmed Dubelcco's modified phosphate-buffered saline (DPBS) and then fixed and permeabilized with 4% formaldehyde (Sigma F8775) and 0.1% Triton X-100 (Serva 37238) in DPBS for 5 min at room temperature. After 5 washing steps, the cells were incubated in blocking buffer for 1 h at room temperature (DPBS containing 2% bovine serum albumin (Sigma A4503), 1% fish gelatin (Sigma G7765), 0.1% Triton X-100, and 5% goat serum (Gibco 16210-064). Then the samples were incubated overnight at 4°C with the anti-TOMM20 antibody (1:250, ab186734, Abcam) diluted in blocking buffer. On the next day, after washing 3× with DPBS, the cells were incubated for 1 h at room temperature with the secondary antibodies (goat anti-rabbit IgG, Jackson ImmunoResearch, 111-005- 003) conjugated with NHS-NC (23), diluted 1:100 in blocking buffer. After washing 2× with DPBS, and 2× with PBS, **Tet-SiR** dye (10 μM in PBS) was added, incubated at room temperature for 60 min, then washed 2× with PBS quickly, and 1× for 2 hours. This was followed by staining overnight with 0.3 μM **tBuTetRhod** in PBS at room temperature. Finally, samples were washed with PBS 2× quickly, and 1× for 2 hours and were imaged with a confocal microscope.

2.9. Microscopy

2.9.1. Fluorescent digital wide field microscopy

Fluorescent wide-field microscopic images of live HEK293T cells in 12-well plates (Greiner Bio-One 665 180; 1 ml/well, as described in 4.3.) were captured using a Biotek Synergy 2 Cytation 3 imaging plate reader with Gen5 software version 3.08 (Biotek Winooski, VT, USA) using a 4x PLFL Olympus objective in red fluorescence (led cube: 523 nm, band pass filter cubes: excitation 530 ± 12.5 nm, emission 590 ± 17.5 nm), green fluorescence (led cube: 465 nm, band pass filter cubes: excitation 485 ± 10 nm, emission 528 ± 10 nm) and bright field channels.

2.9.2. Fluorescent confocal microscopy

Confocal images were acquired on a Leica TCS SP8 microscope using the 488 nm, 552 nm and 638 nm lasers for excitation. The images were taken using a Leica HC PL APO 100x/1.40 oil immersion objective along with Leica PMT and HyD detectors. Multi-colour images were taken using scanning between lines sequences (channel 1: PMT1 detector, channel 2: PMT2 detector, channel 3: HyD detector). Gating in each channel was adjusted for minimal bleed-through between the fluorophores. Live cells were imaged in indicator-free DMEM complete medium (Gibco 21063-029). Fixed cells were imaged in PBS.

The following detection windows were selected for live cell multi-colour experiments: green channel (for the detection of the GFP) 495-550 nm; red channel (for the emission of tBuTetCy3 conjugate) 565-650 nm; far red channel (for the emission of **SiR**) 650-800 nm.

The following detection windows were selected for fixed cell multicolour staining experiments: red channel (for the emission of rhodamine) 565-615 nm; far red channel (for the emission of **SiR**) 675-800 nm. Applying the Huygens Professional software (SVI), we performed deconvolution for image restoration on the recorded multichannel images. The deconvolution was based on theoretical point spread function (PSF). Images were analyzed using Leica Application Suite X and ImageJ software (NIH).

2.10. Cell viability test

An MTT test was carried out to verify nontoxicity of **BICK** and **tBuTetCy3** on HEK293T cells. Cells were transferred into a 48-well plate (Thermo Fisher Scientific, 130187) (40,000 cells/well) and incubated for 20– 24 h at 37 °C in a 5% CO_2 atmosphere. Cells were treated with 1 mM **BICK** or 100 µM tBuTetCy3 or 100 µM, 10 µM, and 1 µM the known toxic drug DOX (doxorubicin hydrochloride, Fluorochem, 021790) for 16 h at 37 °C in a 5% CO2 atmosphere. After the incubation period, supernatants were replaced with 0.5 mg/ml MTT (3-(4,5-dimethylthiazol-2-yl)-2,5-diphenyltetrazolium bromide) solution (in complete DMEM) and incubated for 120 min at 37 °C in the dark. The insoluble formazan crystals were dissolved in 250 µl DMSO. Absorbance was detected at 540 nm using a Biotek Synergy 2 Cytation 3 imaging plate reader with Gen5 software version 3.08 (Biotek Winooski, VT, USA). Viability was expressed as percentage of the readings of untreated control cells.

2.11. NMR spectra

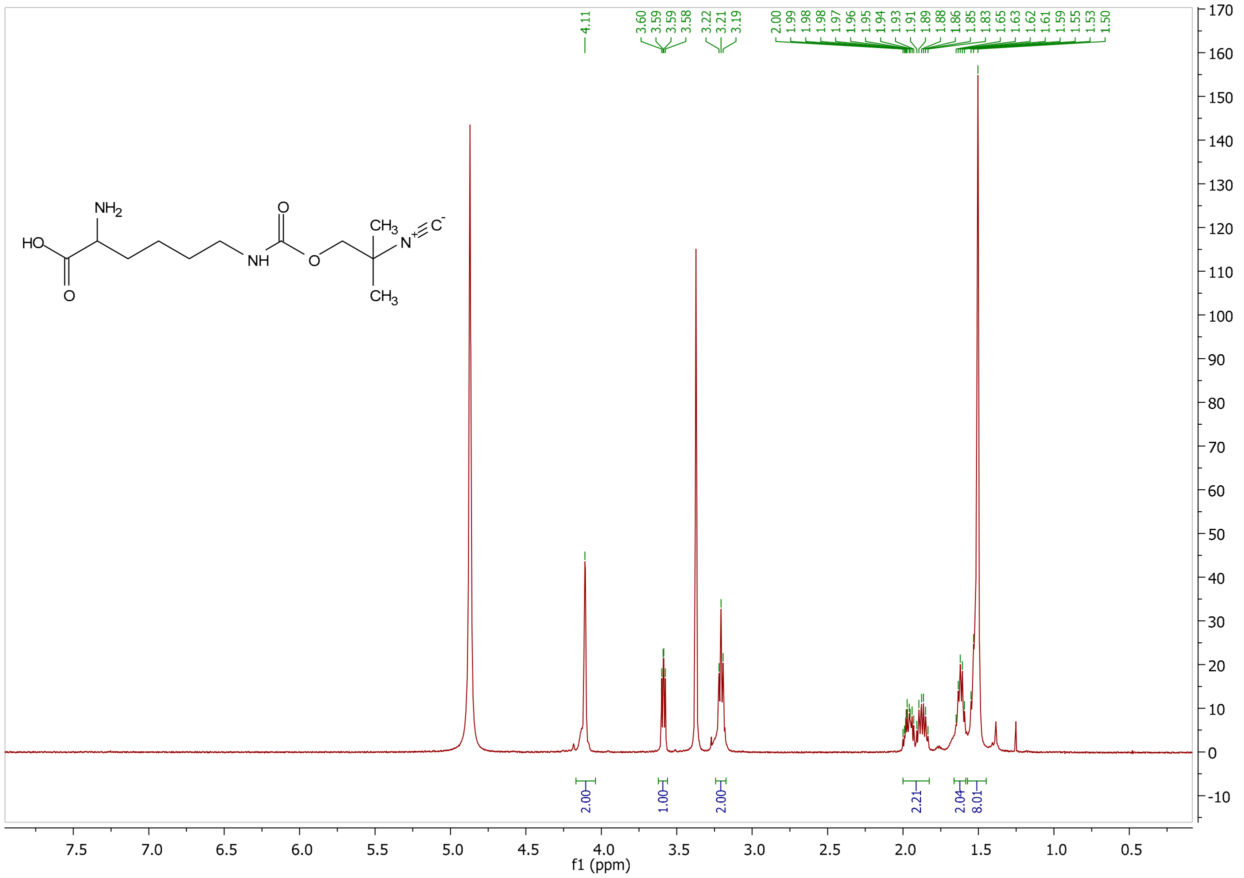

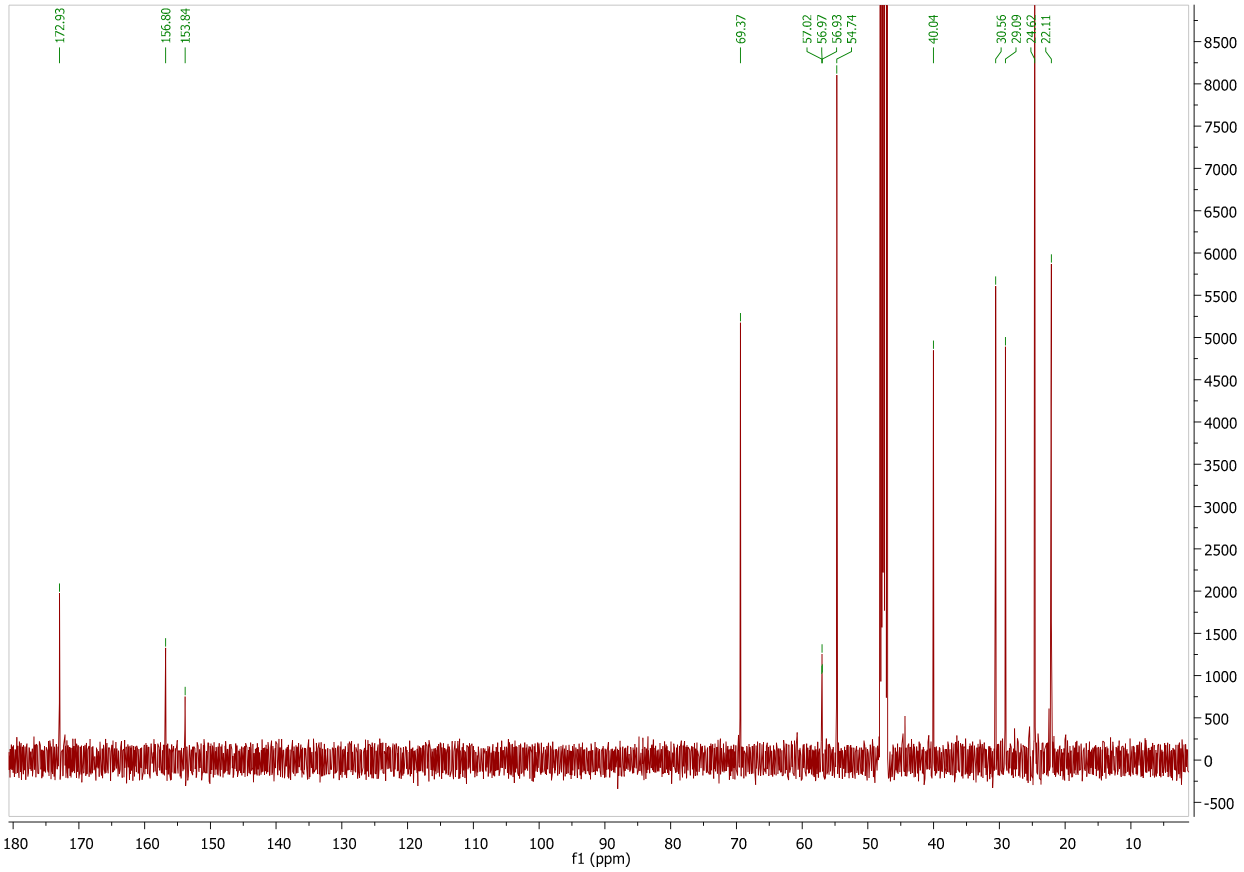

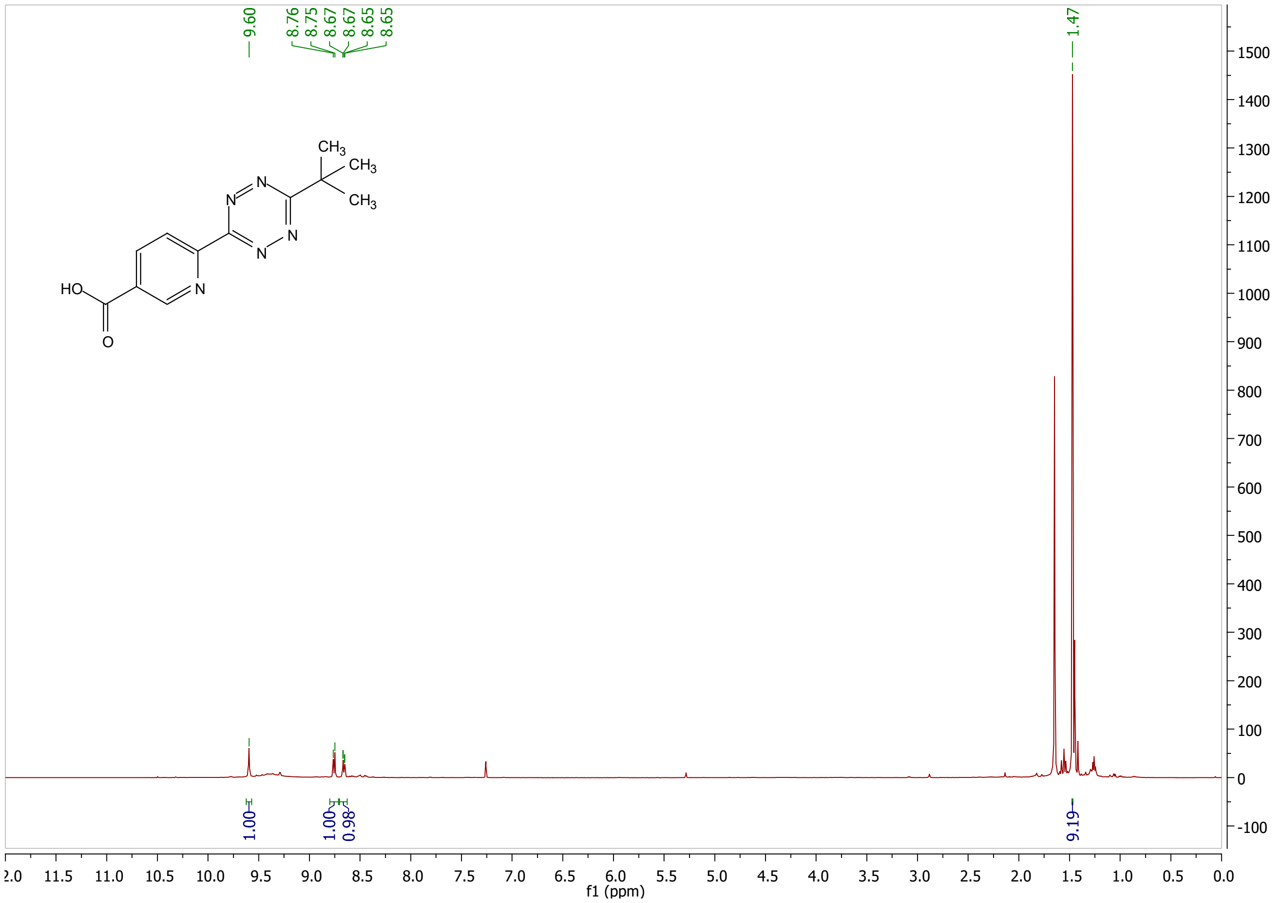
9.60
8.76
8.75
8.67
8.67
8.65
8.65
1.47
CH3
CH3
CH3
N
N
N
N
N
HO
O
1.00
1.00
0.98
9.19
f1 (ppm)

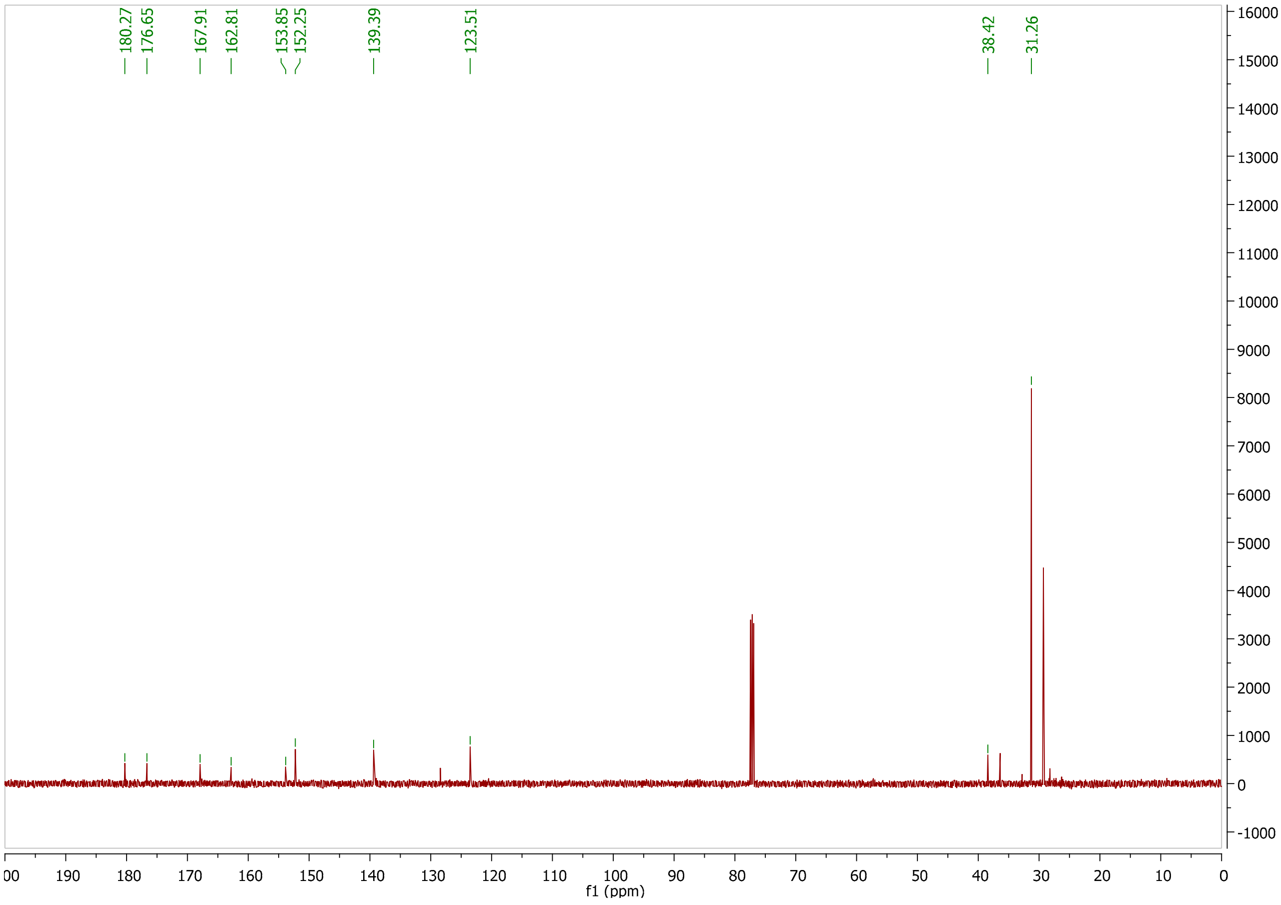
180.27
176.65
167.91
162.81
153.85
152.25
139.39
123.51
38.42
31.26
f1 (ppm)

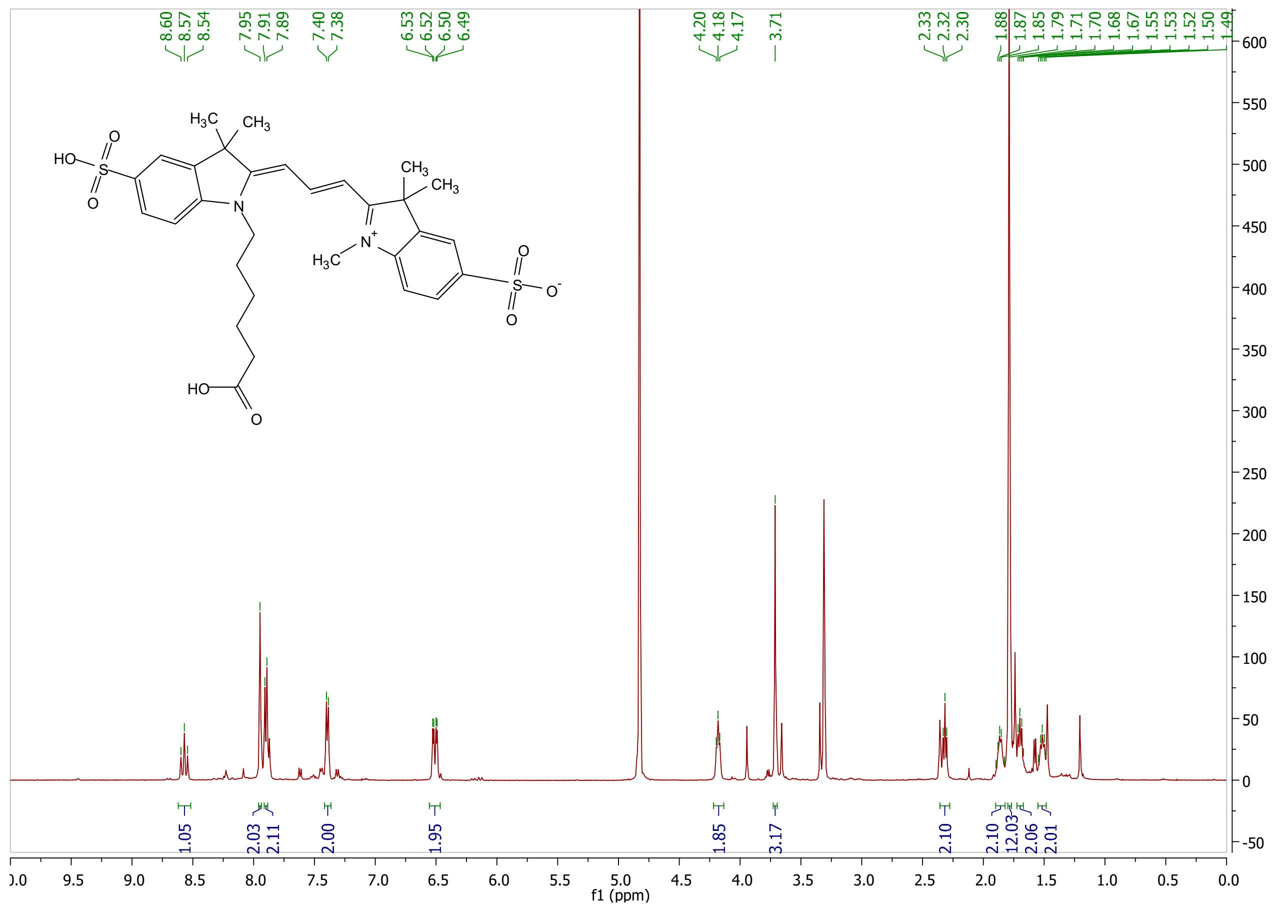
8.60
8.57
8.54
7.95
7.91
7.89
7.40
7.38
6.53
6.52
6.50
6.49
4.20
4.18
4.17
3.71
2.33
2.32
2.30
1.88
1.87
1.85
1.79
1.71
1.70
1.68
1.67
1.55
1.53
1.52
1.50
1.49
1.05
2.03
2.11
2.00
1.95
1.85
3.17
2.10
2.10
12.03
2.06
2.01
f1 (ppm)

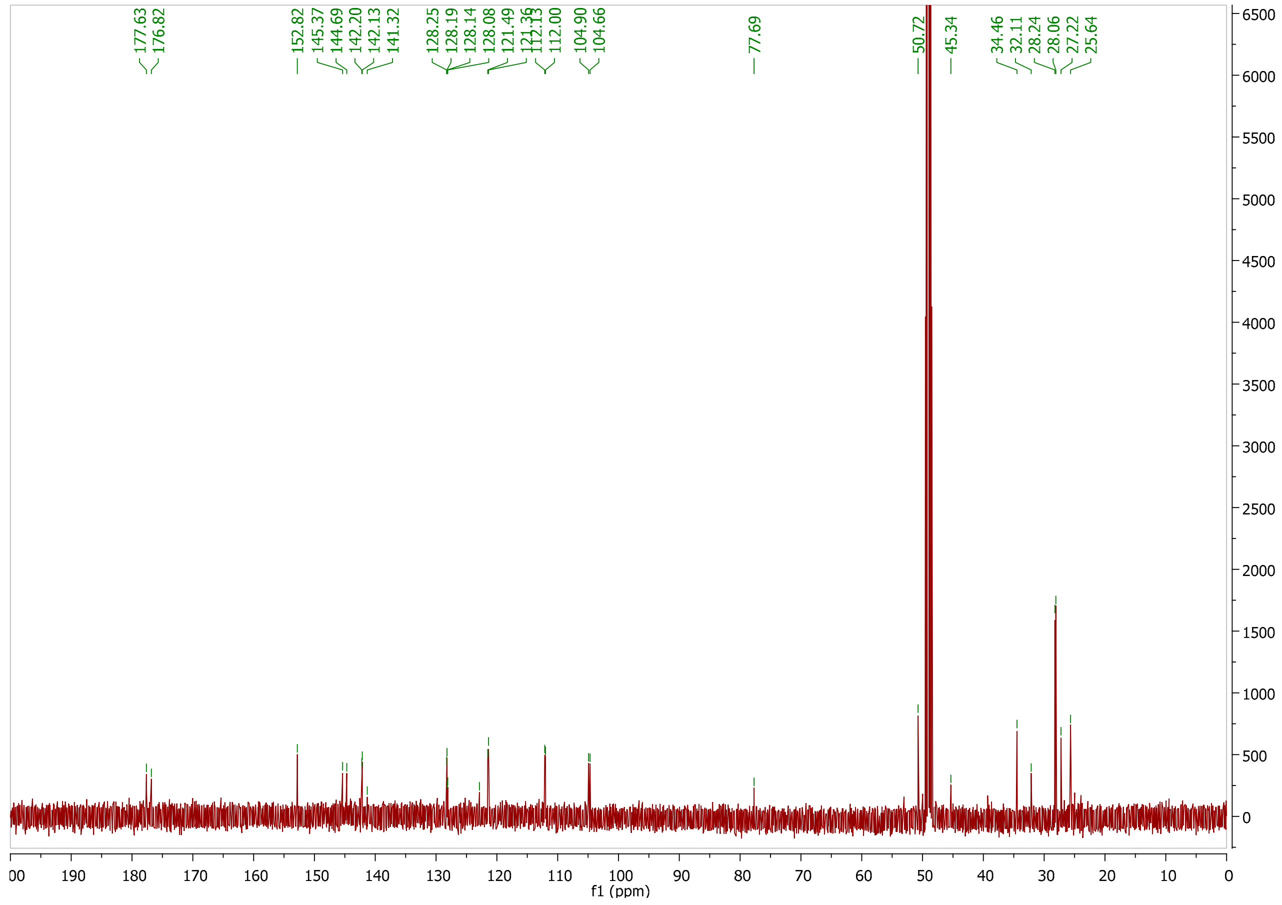
177.63
176.82
152.82
145.37
144.69
142.20
142.13
141.32
128.25
128.19
128.14
128.08
121.49
121.36
121.13
112.00
104.90
104.66
77.69
50.72
45.34
34.46
32.11
28.24
28.06
27.22
25.64
f1 (ppm)

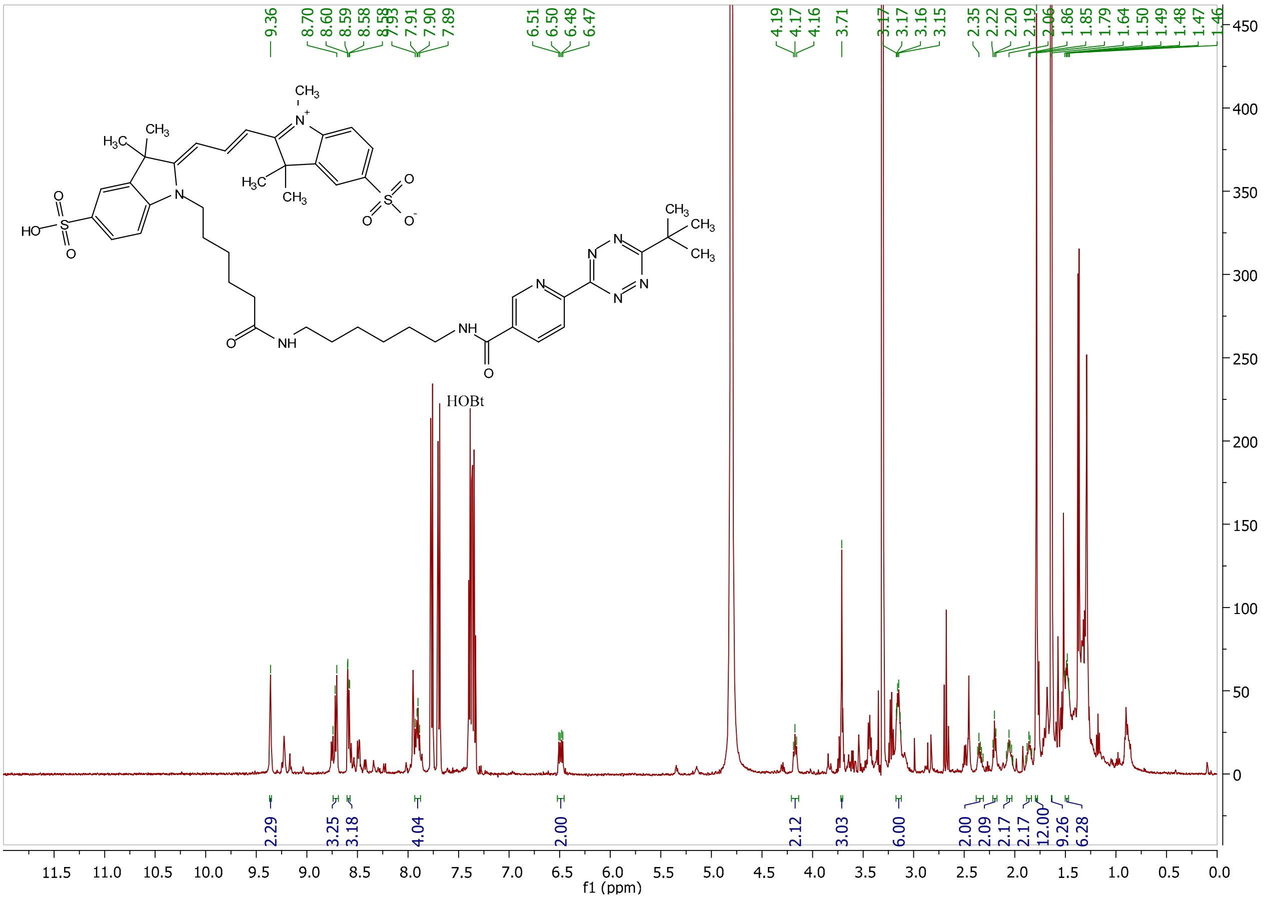
HOBt
f1 (ppm)

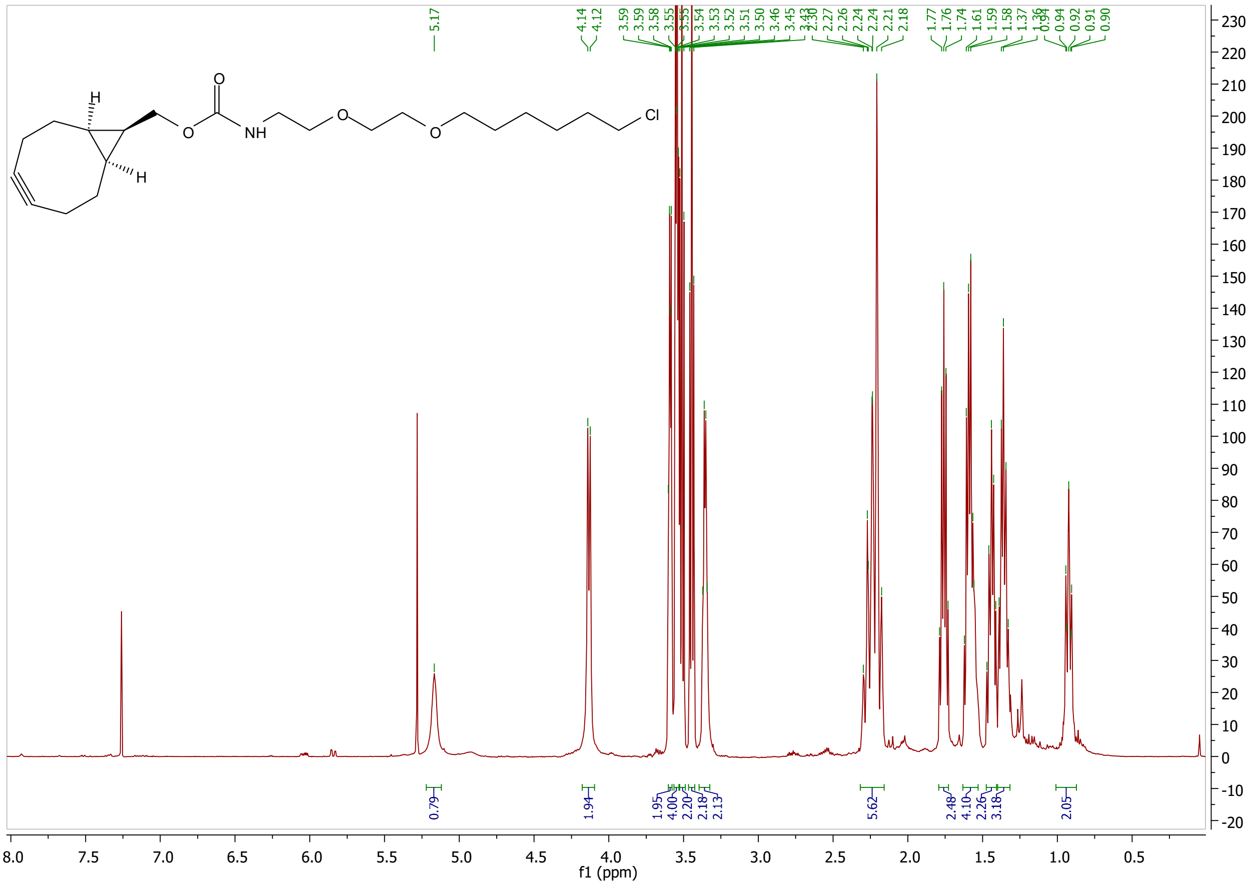
f1 (ppm)

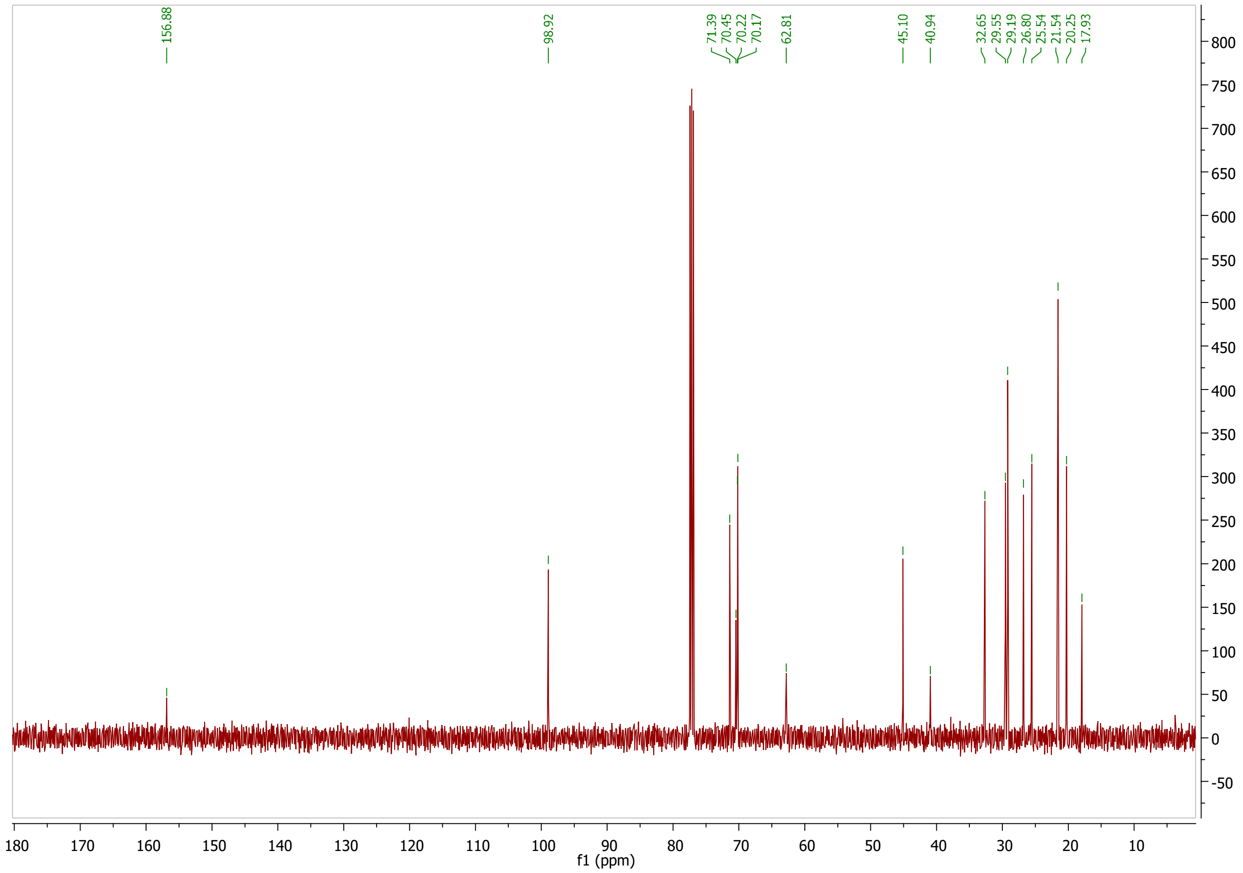
156.88
98.92
71.39
70.45
70.22
70.17
62.81
45.10
40.94
32.65
29.55
29.19
26.80
25.54
21.54
20.25
17.93
f1 (ppm)

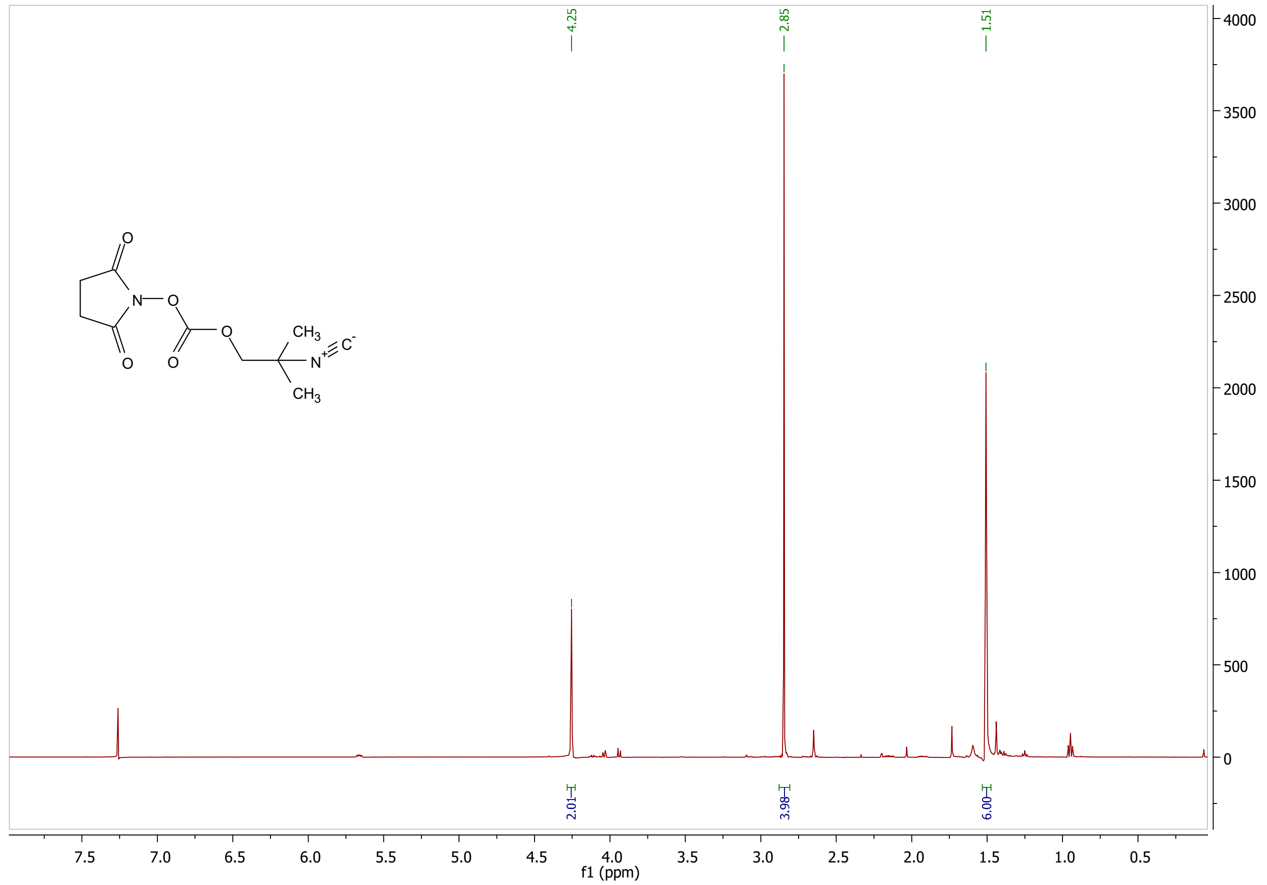
4.25
2.85
1.51
2.01
3.98
6.00
f1 (ppm)

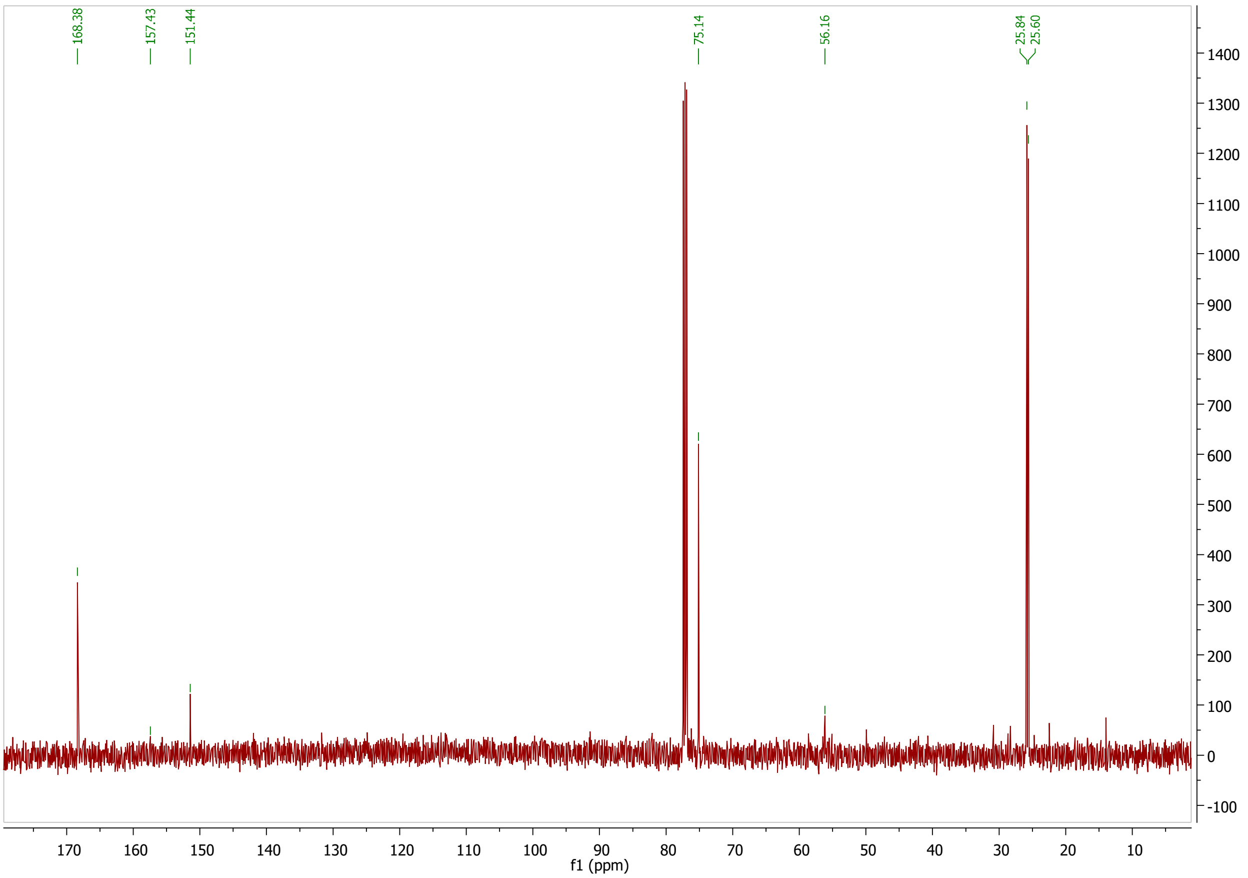

168.38
157.43
151.44
75.14
56.16
25.84
25.60
f1 (ppm)
